# Global-change vulnerability of a key plant resource, the African palms

**DOI:** 10.1038/srep12611

**Published:** 2015-07-27

**Authors:** Anne Blach-Overgaard, Henrik Balslev, John Dransfield, Signe Normand, Jens-Christian Svenning

**Affiliations:** 1 Section for Ecoinformatics and Biodiversity, Department of Bioscience, Aarhus University, Ny Munkegade 114, DK-8000 Aarhus C, Denmark; 2 Royal Botanic Gardens, Kew, Richmond, Surrey TW9 3AB, UK; 3 Landscape Dynamics, Swiss Federal Research Institute, Zürcherstrasse 111, 8903 Birmensdorf, Switzerland

## Abstract

Palms are keystone species in tropical ecosystems and provide essential ecosystem services to rural people worldwide. However, many palm species are threatened by habitat loss and over-exploitation. Furthermore, palms are sensitive to climate and thus vulnerable to future climate changes. Here, we provide a first quantitative assessment of the future risks to the African palm flora, finding that African palm species on average may experience a decline in climatic suitability in >70% of their current ranges by 2080. This suitability loss may, however, be almost halved if migration to nearby climatically suitable sites succeeds. Worryingly, 42% of the areas with 80–100% of species losing climate suitability are also characterized by high human population density (HPD). By 2080, >90% of all African palm species’ ranges will likely occur at HPDs leading to increased risks of habitat loss and overexploitation. Additionally, up to 87% of all species are predicted to lose climatic suitability within current protected areas (PAs) by 2080. In summary, a major plant component of tropical ecosystems and provider of ecosystem services to rural populations will face strongly increased pressures from climate change and human populations in the near future.

The palm family (Arecaceae) is represented by 65 species on continental Africa^1^. African palms differ from those in other regions by a comparatively low species richness^2^, but their commonness make them important providers of ecosystem services to both rural communities and commercial agriculture^3^. They also serve as food source for many animal species, e.g., chimpanzees, baboons, elephants and duikers^4^. Wild populations of African palms are under pressure from over-exploitation^5^ and some already have declining populations due to unsustainable utilisation^6^,^7^. To exacerbate the pressures on palm populations, losses of important palm habitats such as tropical rain forests are steadily increasing^8^. These risks are likely to increase in the future, given the predicted growth in human populations across Africa^9^. The climate sensitivity of palms is well known^10^,^11^,^12^, and long-term historical climate change effects are still discernible in their present-day diversity patterns^2^,^13^, suggesting that future climate changes pose a threat with potentially long-lasting effects. Thus, palms and their associated ecosystem functions and services may be at risk not just from direct pressures from exploitation and land-use, but also from climate change. Given the socioeconomic and ecological importance of the palms it is essential to understand how future climate change and increasing human populations will affect African palms and to evaluate the potential of the existing network of protected areas across Africa to mitigate these pressures.

Using a comprehensive data set for 40 African palm species with sufficient unique occurrence records (Supplementary Table S1) we assessed the intensity of future threats from climate change and human impact to African palms between a baseline period (1960−1990) and three future time intervals centred on 2020, 2050 and 2080. We predicted the possible consequences of climate change using species distribution models (SDM) by estimating the extent to which palm species are projected to lose (‘local climate losers’) or gain (‘local climate winners’) climate suitability within their predicted current range, as well as gain climatic suitability by migration (‘immigrant climate winners’) (Methods; Supplementary Methods). Future increases in CO_2_ concentrations may change species-climate relations^14^. We included this potential effect in the modelling by applying two sets of precipitation data; one rescaled to account for the CO_2_ effect and one with raw values (Methods; Supplementary Methods). We also estimated potential future risks to palms from land-use intensification and unsustainable harvesting by estimating the average projected HPD within each species range and further quantified the spatial overlap in climate change and HPD stress. Finally, we also assessed the potential buffering role of the cross-African conservation area network by evaluating the ability of PAs across Africa to retain climatic suitability for palms by the end of the 21^st^ century (Methods). We made assessments for two dispersal scenarios, a pessimistic no-dispersal scenario and an optimistic, but still realistic scenario with dispersal up to 100 km beyond the current range (Supplementary Methods). These scenarios reflect the limited dispersal capacity of many palms^11^,^15^ as well as known migration rates for tropical trees^16^.

## Results and discussion

**Figure 1:**
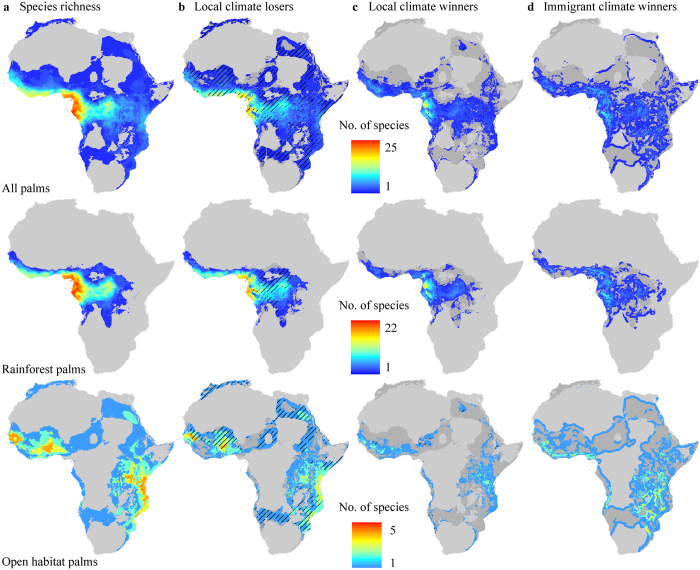
Current palm species richness patterns and three measures (‘local climate losers’, ‘local climate winners’ and ‘immigrant climate winners’) of the projected impacts of climate change for year 2080. (**a**) Spatial variation in species richness (number of species per grid) of all palms (*n* = 40), rainforest palms (*n* = 27) and open-habitat palms (*n* = 11). (**b**) Spatial overlap of the number of species projected to lose climate suitability in a given area. (**c**) Spatial overlap of the number of species projected to gain climate suitability in a given area. (**d**) Number of species per grid projected to gain climate suitability in a given area outside their current range at distances up to 100 km. Values for (**b–d**) were computed as arithmetic means across two species distribution models, three global circulation models and three gas emission scenarios for the +CO_2_ scenario (Methods). The light grey areas in all panels indicate the absence of palms, while the darker shade grey denotes the predicted baseline range limit of a particular palm group. The black hatched areas in b and c indicate where climate losers or winners, respectively, constitute 80−100% of the total species pool in a given area. The spatial richness patterns in relative palm suitability changes were consistent over time periods, but the proportions of local losers increased with time (see Supplementary Figs S1−S2). The map was created in ArcGIS 9.3 (ESRI, Redlands, CA, USA).

Our results show that the African palms will be highly sensitive to future climate change. We predicted climate suitability losses across almost the entire range where palms occur in Africa, while climate suitability increases were only predicted for fewer species in smaller areas (Fig. 1; Supplementary Figs S1-S2). The largest numbers of local climate losers by year 2080 were concentrated in the coastal zones of the Guineo-Congolian Floristic Region with up to 21 species in the most exposed site in Gabon, corresponding to 87% of the local palm species pool. This was driven by the high number of rainforest palm species losing climate suitability in this region. In contrast, most open-habitat palms were projected to lose climate suitability in West Africa and coastal East Africa (Fig. 1). Absolute and proportional potential losses of palm species did not coincide geographically (Fig. 1). We found high proportions (80–100%) of species losing climate suitability by 2080 across large tracts of land in Africa, extending far beyond the regions of highest richness of climate loser species. In particular, our results show a high proportion of rainforest species losing climate suitability in Central Africa and open-habitat palms losing climate suitability in the Mediterranean, Sudano-Sahelian, Zambezian, Somalian and parts of Saharan zones (Fig. 1b). These marked differences between areas harbouring high species richness of climate losers and areas with the highest proportion of species losing climate suitability indicate that many areas with relatively few palm species are at risk of suffering a complete or near-complete loss of this key functional group of wild plants. Such losses may not only have disruptive effects on ecosystem functioning^17^, but also highly negative consequences for local people depending on palm products for their livelihood^1^. The estimated high sensitivity of African palms to climate change is supported by field studies^18^,^19^.

For all groups of palms (see methods), we projected on average a decline in climate suitability in >70% of their current predicted ranges by 2080. However, we found that many palms could potentially track suitable climate space by dispersing within 100-km distance of their current range margins (Table 1, Fig. 1). Allowing for such colonisations we found the highest gain in climate suitability in west-central Africa for palms overall and for rainforest palms, but in south-eastern Africa for open-habitat palms (Fig. 1d). When accounting for potential colonisation the average loss of climate suitability across all palm species and open-habitat palms were almost halved, with a slightly weaker effect on rainforest palms (Table 1). However, palm species should on average expand into new regions at ~1000 meter/year, corresponding to top-end known migration rates for trees^16^,^20^ to make these potential suitability gains a reality. It seems unrealistic that such high migration rates would be possible in general, especially because many palms are thought to have poor dispersal capacity^11^,^15^ and because range shifts^21^ may be impaired by habitat fragmentation with novel anthropogenic habitats constituting dispersal barriers^22^. More broadly considered, strong disequilibria in distribution, abundance, and population structure of many plants must be expected under the short time scales for 21^st^ century climate change^23^.

**Figure 2:**
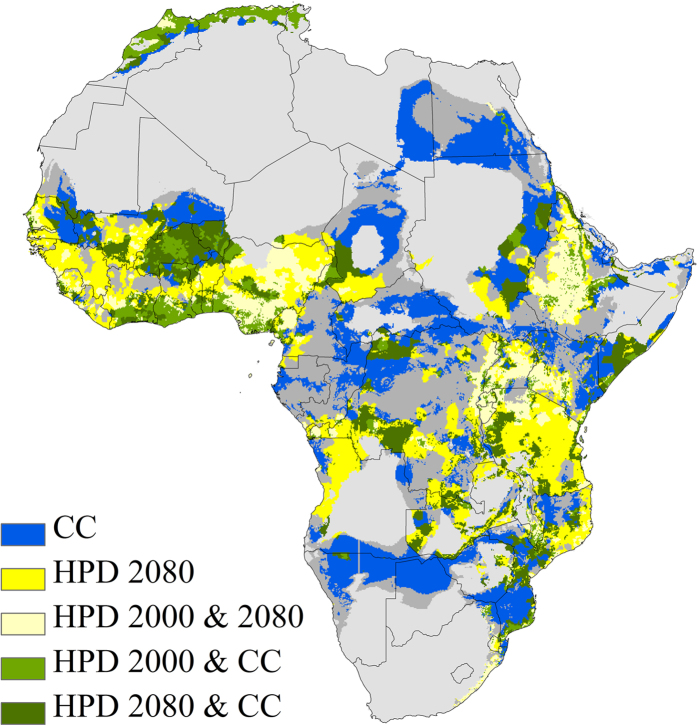
Vulnerability of climate change and human population density increases: spatial overlap between areas with the highest proportion ‘local climate losers’ in 2080 and human population density exceeding the average continental-wide human population density for year 2000 (CON-HPD). Areas with the highest proportion of ‘local climate losers’ are defined as the grids with 80–100% of all palms losing climate suitability within their current range by 2080 for the +CO_2_ scenario (CC; blue areas). Human population density (HPD) projected for 2080 is mapped (dark yellow areas) for grid cells that exceed CON-HPD (≥50.14 persons/km^2^). Light yellow areas denote where HPD for both year 2080 and year 2000 exceeds CON-HPD (HPD 2000 & 2080). Light green areas marks the spatial overlap between HPD 2000 & 2080 and areas with the highest proportion of palm species losing climate suitability by 2080. Areas where high levels of HPD for 2080 coincide with the highest proportion of palm species losing climate suitability by 2080 are marked in dark green. HPD and CC are only mapped within the range of the palm family (dark grey areas). The map was created in ArcGIS 9.3 (ESRI, Redlands, CA, USA).

The focus on single threats to biodiversity may be inadequate due to synergistic effects between different pressures^24^. Land-use and climate changes have been ranked as the biggest threats to biodiversity in the 21^st^ century^25^; hence we assessed their combined effects on African palms. Land- use is related to HPD, and we set a threshold for high risk of habitat loss and overexploitation for palms at the continental-wide average HPD for year 2000 (CON-HPD; 50.14 people/km^2^). Most natural land cover classes have mean HPD below this threshold, while most anthropogenic land cover classes and especially those in which most palm species would not be able to maintain populations have higher HPD (Supplementary Methods; Supplementary Table S2). Our results show that 42.5% of the 10 × 10 km grid cells projected to have the highest proportion of climate loser species (80–100%) in 2080 have projected HPD for year 2080 exceeding the CON-HPD benchmark. This was the case for large regions in northern and western Africa as well as multiple smaller areas in eastern Africa (Fig. 2). Importantly, one third (33.7%) of the cells in these areas already today have HPD exceeding the high HPD threshold (Fig. 2). Palm populations in these areas may already be under pressure from human activities today and will in the future be in double jeopardy due to the increasing HPD and decreasing climatic suitability. However, there were also large areas of non-overlap between the two threats, underscoring that focussing on single pressures is insufficient for forecasting future changes in biodiversity and associated ecosystem services^24^.

**Figure 3:**
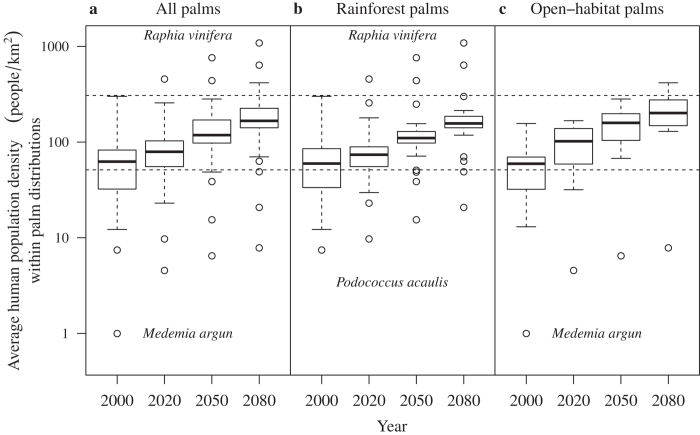
Average human population density within areas of palm distributions. Average human population density (on log_10_ scale) within 100-km-dispersal ranges of (**a)** all palms (*n* = 40), (**b**) rainforest palms (*n* = 27) and (**c**) open-habitat palms (*n* = 11) for the +CO_2_ scenario (Methods) for four time periods in the 21^st^ century. The upper and lower dotted lines mark population density in the densest populated country in Africa in year 2000 (Rwanda; 307 people/km^2^) and the continental-wide average human population density in year 2000 (50.14 people/km^2^), respectively. The most extreme outlier species for each group and time slice at high HPD are *Raphia vinifera* (panel a,b) and at low HPD *Medemia argun* (**a,c**) and *Podococcus acaulis* (**b**). Values are computed as arithmetic means of all model combinations of two species distribution models, three global circulation models and three gas emissions. The horizontal black bar is the median for each group per time period. The box indicates the interquartile range and whiskers extend to the most extreme data point, which is no more than 1.5 times the interquartile range from the box, while more extreme values are displayed as open circles.

For all groups of palms the projected average HPDs within species’ ranges were estimated to increase over time under both dispersal scenarios (Fig. 3; Supplementary Fig. S3). Notably, by 2080 the average HPD would potentially exceed CON-HPD for 92.5% of all palm species, for 92.6% of rainforest species and for 90.0% of open-habitat species. By the end of the century, three palm species were projected to occur under HPDs higher than that of Rwanda (Fig. 3), the currently most densely populated country in Africa (307 people/km^2^ in year 2000). Such high HPD across complete species ranges is worrying as it does not seem likely that wild palm populations will be able to persist under such conditions, as these have led, amongst other biodiversity threats, to severe land degradation, deforestation, soil erosion and unsustainable ground water extractions in Rwanda^26^. Similar changes have taken place in Uganda, driven by demand for additional agricultural land due to rapid human population increases, causing the loss of forest and woodland habitats and putting additional pressure on local threatened plants such as the palm *Raphia farinifera*^27^. Although it has been noted that some palms are spared during deforestation^1^, habitat degradation and fragmentation may directly impair survival and recruitment and also influence the viability of palm communities via negative effects on pollinators or dispersers^28^. Additionally, palms are highly utilized resources in many rural communities, meaning that the demand for palm products is bound to rise with human population increases^29^. Furthermore, community-level assessments of coping strategies to climatic extremes show that not only are African ecosystems stressed by unsustainable management and land-use change, but that climate change exacerbates the impact of these stressors by making rural communities even more reliant on non-timber forest products^30^ such as palms for subsistence, creating incentive for over-exploitation of palm resources^31^. Over-exploitation of wild palm populations is already detectable under current human population densities in parts of Africa^6^. In Guinea, *Borassus aethiopum* populations are threatened by extinction due to destructive palm wine harvesting methods^6^. Similarly, palm sap harvesting of *B. aethiopum* and *Raphia hookeri* in the Ivory Coast have been found unsustainable, particularly in villages with dense human population^7^. Further south in Botswana, *Hyphaene petersiana* populations are likewise utilized for their sap using unsustainable methods such as burning and felling, resulting in unstable population structure (due to declining densities of certain groups of palm individuals classified based on diameter at breast height) and reduced regeneration of the species from seeds^32^.

Overall, lower median average HPD within palm distributions was found for rainforest palms compared to open-habitat palms for 2080 (Fig. 3, Supplementary Fig. S3); however, some rainforest palms were projected to experience very high HPDs across their future ranges by the end of the century, e.g., *Raphia vinifera* (confined to southern Nigeria) with up to nearly 1200 people/km^2^. In contrast, the open-habitat palm *Medemia argun*, an oasis palm of the Nubian Desert, was estimated to occur on average under HPDs of 8 people/km^2^ even by year 2080 (Fig. 3). Although this is low relative to other palms (Fig. 3), this species occurs in a harsh desert environment where excessive utilization is already a serious threat^33^. We used average HPD across species ranges as a summary measure of anthropogenic impact. This means that palms in certain areas of their ranges may experience higher HPD, because HPD is often unevenly distributed across the landscape. However, palms may not be affected just in high HPD areas due to the local effects that high HPDs will have on palms; palm populations may also be affected in less densely settled areas, because human pressures often extend beyond areas of human habitation^34^. For example, in the Okavango Delta in Botswana, local villages have utilised *H. petersiana* for commercial basket weaving, but the high demand for palm material has led to depleted stands near villages, causing harvesting to be carried out further and further away^35^.

**Figure 4:**
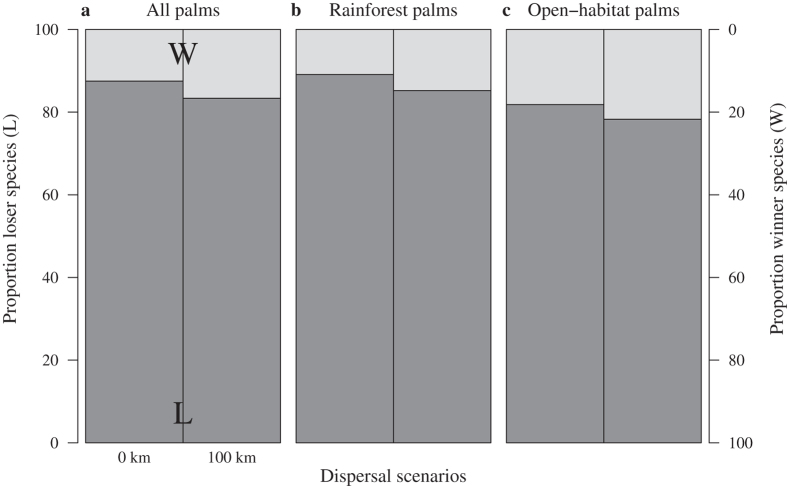
Proportion of palm species losing or gaining climate suitability within the protected area network in Africa, projected for 2080. Proportion of (**a**) all palms (n = 40), (**b**) rainforest palms and (**c**) open-habitat palm species projected to lose (losers; **L**) or gain (winners; **W**) climate suitability in the protected areas across continental Africa by 2080 for two dispersal scenarios, no-dispersal (0 km) and 100-km-dispersal (100 km) for the +CO_2_ scenario (Methods). Values computed as arithmetic means of all model combinations of two species distribution models, three global circulation models and three gas emissions.

Protected areas (PAs) represent cornerstone conservation tools to protect species and their habitats. In light of the overall threats from climate change and the increasing human population on African palms, our results unfortunately points to the fact that PAs in Africa where palms occur potentially have a relative low resilience to future climate change. Climate suitability of palms in the protected area network across Africa was projected to decrease markedly by the end of the century, with a high proportion of species losing climate suitability within the existing PAs (87.5% of all palms; 89.1% of rainforest palms alone, and 81.1% of open-habitat palms alone; Fig. 4). The low effectiveness of the African PAs in the face of climate change has also been noted for other groups of organisms in Africa^36^. We did, however, find some buffering effects to be provided by the PAs in Africa, reflecting immigration of palm species into protected areas (Fig. 4, Supplementary Table S3). Overall, it is problematic that the protected area network as it stands today will not be an effective safe haven for future African palm populations, and not just from the perspective of maintaining palm diversity, but also considering the ecological integrity of the PAs.

The potential loss of the key plant resource that palms constitute due to climate change and human impacts is likely to affect both natural ecosystems^17^ and rural human populations. Notably, it will threaten livelihoods in African rural communities relying on palm products for subsistence^3^, as well as for frugivorous birds and mammals that depend on palm fruits and seeds for their diet^37^. To alleviate some of the consequences of climate change and human population increases in Africa, improvement of food security, sustainable management practices and climate change mitigation strategies need to go hand in hand^38^,^39^. Notably, both land-sharing and land-sparing strategies should be considered^40^,^41^. In terms of land-sharing, there is a need to promote more sustainable land-use practices such as agroforestry, which is a traditional land-use system in Africa where individuals of important trees such as palms or other key plants are maintained within cultivated fields^42^,^43^. These parkland agroforestry systems serve as a source of income, but may also play a crucial role in climate mitigation actions as such parklands can be considered as carbon sinks with high carbon sequestration potential^39^. At the same time improved land-sparing strategies will likely also be essential, as it seems unlikely that all palms (and other species) can be integrated into agroforestry and other agricultural systems. Hence, a thorough investigation into the current and future efficiency of the existing protected area network in Africa will be important^44^,^45^. Although protected areas may help some species tracking climate change^46^, they are static systems and as such may be limited in their ability to remain safe havens for current species assemblages in the future, as is indicated by the current study and others^36^,^47^,^48^. Notably, an expansion of the protected area network may be needed to increase their effectiveness under future climate change. To overcome the risk from climate change, assisted migration, human-mediated colonisations beyond species’ current range to areas where they will have a higher probability of survival under future climate conditions^49^, may be needed for both land-sharing and land-sparing strategies. Notably, for highly threatened African palms this could be the only way of securing their survival given their limited dispersal abilities^11^,^15^.

To account for the uncertainty inherent in forecasting species distributions under different climate projections, we employed ensemble modelling. It has been documented that using alternative models can provide variable forecasts and using single modelling approaches may therefore lead to misguided interpretations^50^. The uncertainties arise not only from the SDM algorithms, but also in the choice of global circulation models (GCMs) and gas emission scenarios (SRES)^51^,^52^. Ensemble modelling provides a way to address this variability, as the central tendency in such forecasts have been shown to provide more robust predictions^51^. For the current study we addressed the potential uncertainty in forecasting palm distributions by selecting two SDM algorithms (see Methods for details), which have been found to both have better predictive capacity for palm species distributions in Africa than other methods^11^,^13^. In addition, we selected future climatic data derived from three different GCMs for three SRES, together representing a variety of assumptions about climate system and future green-house gas emissions, with the latter encompassing a range of potential future demographic, social, economic, technological and environmental developments (Supplementary Methods)^53^. Sensitivity analyses show that the reported results are robust to a number of key uncertainties. Notably, they showed only slight differences among the different combinations of SDM, GCM and SRES with the most conspicuous differences noticeable between the SDM algorithms (Supplementary Figs S4-S6), as has also been reported in other studies^52^. The different CO_2_ scenarios did not show large differences when averaged across species for the ensemble forecasts. However for the majority of species there was a decrease in overall climate suitability loss for the +CO_2_ scenario compared to the −CO_2_ scenario (mean difference in climate suitability loss: 11.23% ± 12.93 for all palms, 11.24% ± 14.51 for rainforest palms and 9.76% ± 9.12 for open-habitat palms), with greater differences for some individual species. This may indicate that if increased CO_2_ concentrations indeed enhances water-use efficiency in palms, the consequences may be species-specific and to some extent also localized. It has also recently been shown that CO_2_ effects on vegetation transitions may be abrupt at certain localities, but become weaker when averaged across the African continent^54^. The modelled CO_2_ effect (+CO_2_) caused the estimated climate change impacts to be less severe for most palms, i.e., predicting a lower proportion of climate losers (Table 1), similar to findings for tropical South American plant species responses to climate change^55^. Our approach to rescaling climate predictors to better represent how plant functionally perceive them offer a relatively simple way to account for CO_2_ effects on water-use efficiency in climate change studies. However, the methodology should ideally be tested and refined by physiological experiments on tropical plants.

Our study presents a first quantitative continent-wide assessment of the potential risk from future climate change and its interaction with increased human populations for a keystone group of wild plants, the African palms. Given the role of palms in African and other tropical ecosystems and as essential source of income and resource for daily needs for many rural communities worldwide, our results call for attention as to how this important plant family and its ecological functions can be conserved for future generations. We suggest that both land-sharing and land-sparing strategies need to be considered, notably improved agroforestry practices with better integration of wild palm species, an expanded network of protected areas, and, for both, the use of assisted colonization to help species track the climate changes.

## Methods

We fitted species distribution models (SDM) to locality records for 40 African palm species (Supplementary Table S1) to three temperature and three water balance-derived variables at 10 × 10 km resolution for the baseline period (1960−1990). To account for the potential effect of the changed water-use efficiency of plants under different CO_2_ levels^56^, we subsequently projected all models onto two sets of future climate scenarios, one set accounting for the potential water-use efficiency in plants under increased CO_2_ levels (+CO_2_) and a set of unmodified variables (−CO_2_) (Supplementary Methods; Supplementary Fig. S7, Table S4-S5). To account for the uncertainty inherent in climate projections we applied an ensemble approach^51^, using two species distribution modelling algorithms (Maxent and Generalised Boosting Model), as well three different future gas emission scenarios A1B, A2A and B2A (SRES)^53^ given by three different coupled Atmosphere-Ocean global circulation models (GCM) namely CCCMA (CGCM2/CGCM3), CSIRO (MK2/MK3.1) and HADCM3 (HACCPR/UKMO) for three future time periods centred on 2020, 2050, and 2080. These future climatic data sets represented a range of potential climatic conditions (Supplementary Methods; Supplementary Figs S8-S9).

We made assessments for two dispersal scenarios, a pessimistic no-dispersal scenario and an optimistic, but still realistic scenario with dispersal up to 100 km beyond the current range (Supplementary Methods). We refrained from using the widely used, but unrealistic full dispersal scenario. An alternate approach to account for species individual dispersal abilities would have been to apply more mechanistic or process-based models which incorporate life-history traits, population dynamics and seed dispersal. However, given the low dispersal abilities of palms we believe that providing a potential dispersal range between 0 and 100 km are sufficient for the addressed questions as these scenarios reflect the limited dispersal capacity of many palms^11^,^15^ as well as the known migration rates for tropical trees^16^.

We assessed the impact of climate change by calculating the change in climatic suitability as the difference in climatic suitability between the baseline and future conditions for all 40 species across all model combinations (SDM × GCM × SRES) for both CO_2_ scenarios. Within species’ current predicted range, we subsequently summed the number of species per 10 × 10-km grid that were predicted to experience negative change in climatic suitability (‘local climate losers’) or alternatively positive change in climatic suitability (‘local climate winners’) between future and current conditions. Species were considered ‘losers’ or ‘winners’ regardless of the magnitude of the change in climatic suitability and could be counted as winners in one grid cell and losers in another grid cell. In addition, we summed the number of species per 10 × 10-km grids that were predicted to gain climatic suitability outside their current predicted range, but no further than 100 km from their current range margins (Supplementary Methods, Supplementary Fig. S10).

We examined the impact of human population density change over time for each species by weighting time-specific human population density maps (Supplementary Fig. S11) by the proportion of climate suitability per grid cell given by the species-specific SDM models within each dispersal mask for all combinations of models (SDM × GCM × SRES × CO_2_-scenario) (Supplementary Methods).

We assessed the impact of climate change on African conservation areas by estimating the number of species gaining or losing climate suitability within the existing protected area network in Africa in the future. We did so by matching the climate suitability given by the species-specific SDM models with the proportion of grid cells that is covered by the current conservation area network across Africa (Supplementary Methods, Supplementary Fig. S12). This was done in a cell-by cell manner for each combination of models (SDM × GCM × SRES × CO_2_-scenario) and for each time period (baseline, 2020, 2050 and 2080). Changes in conserved climate suitability between the baseline and future time periods were calculated for each species (Supplementary Methods).

We performed all assessments for all 40 palms as well as for strict rainforest and open-habitat species, separately (Supplementary Methods). These two groups of African palms exhibit divergent spatial richness patterns (Fig. 1a), reflecting their differing climate affinities and resulting relations to past and present climate^13^. Consensus results for all computations were obtained by calculating arithmetic means across all model combinations (SDM × GCM × SRES) for each time period and CO_2_-scenario. All results provided in the main text are for the +CO_2_-scenario for year 2080 unless other information is given.

Species distribution models using Generalised Boosting Model were computed in R^57^ using the package BIOMOD version 1.1.7^58^. For Maxent we used version 3.3.3k^59^ (Supplementary Methods; Supplementary Table S6). All GIS analyses were done using Python 2.5 and ArcGIS 9.3 (ESRI, Redlands, CA, USA).

## Additional Information

**How to cite this article**: Blach-Overgaard, A. *et al*. Global-change vulnerability of a key plant resource, the African palms. *Sci. Rep*. **5**, 12611; doi: 10.1038/srep12611 (2015).

## Supplementary Material

Supplementary Information

## Figures and Tables

**Table 1:** Overall climate suitability loss for all, rainforest and open-habitat palms.

Palm groups	2020		2050		2080	
	−CO_2_	+CO_2_	−CO_2_	+CO_2_	−CO_2_	+CO_2_
All	55.42 (±14.50)	53.92 (±15.11)	64.59 (±14.71)	62.17 (±15.63)	73.93 (±13.07)	**71.24 (±14.33**)
	19.10 (±30.36)	16.89 (±31.06)	31.60 (±31.79)	28.07 (±32.61)	45.86 (±30.69)	**41.66 (±32.18)**
Rainforest	54.19 (±13.27)	52.56 (±13.75)	64.17 (±14.02)	61.54 (±15.05)	74.36 (±12.37)	**71.48 (±13.72)**
	20.17 (±21.55)	17.81 (±21.83)	34.38 (±23.80)	30.55 (±24.61)	49.80 (±22.86)	**45.39 (±24.31)**
Open-habitat	58.05 (±18.57)	56.81 (±19.45)	66.06 (±17.99)	64.05 (±18.78)	73.74 (±16.15)	**71.53 (±17.42)**
	17.19 (±48.55)	15.40 (±49.92)	26.69 (±48.74)	23.95 (±49.90)	38.41 (±46.48)	**35.00 (±48.55)**

Group average (±standard deviation) of the proportion (%) of climate suitability loss for all palms, rainforest palms and open-habitat palms: For each palm group two values are given: (1) (top row per group) proportion of climate suitability loss within current predicted ranges, and (2) (bottom row per group) overall climate suitability loss (%) when allowing for colonisation within the 100-km margin surrounding the predicted current range for three time intervals and two CO_2_ scenarios (see Methods) in the 21^st^-century. The latter was computed as the number of cells losing climate suitability within the predicted current range of palms minus the number of cells gaining climate suitability within the 100-km margin of a given species out of the total number of cells in a given species’ predicted current range All values were computed as arithmetic means across all model combinations of two species distribution model algorithms, three global circulation models and three gas emissions. Values generally referred to in the text are for the +CO_2_ scenario projected for 2080 which is here marked in bold.
