# Supplementary material for: Global-change vulnerability of a key plant resource, the African palms
Source: Sci Rep. 2015 Jul 27;5:12611. doi: 10.1038/srep12611 (PMC4515823; doi:10.1038/srep12611)
Supplement: Supplementary Information [file srep12611-s1.pdf]

Supplementary information

Global-change vulnerability of a key plant resource, the African palms

Anne Blach-Overgaard[1*], Henrik Balslev[1], John Dransfield[2], Signe Normand[1, 3] & Jens-Christian Svenning[1]

[1]Section for Ecoinformatics and Biodiversity, Department of Bioscience, Aarhus University, Ny Munkegade 114, DK-8000 Aarhus C, Denmark.

[2]Royal Botanic Gardens, Kew, Richmond, Surrey TW9 3AB, UK.

[3]Landscape Dynamics, Swiss Federal Research Institute, Zürcherstrasse 111, 8903 Birmensdorf, Switzerland.

[*]Correspondence: Anne Blach-Overgaard, Department of Bioscience, Aarhus University, Ny Munkegade 114, 8000 Aarhus C, Denmark, email: anne.overgaard@bios.au.dk

Supplementary methods

African palm data

We assembled a comprehensive, geographical database for African palms including > 5500 palm specimens. The occurrence data came primarily from herbarium collections from the Royal Botanic Gardens, Kew; the National Botanic Garden of Belgium; the National Herbarium Nederland; the Missouri Botanical Garden; the National Herbarium of Namibia; and herbarium collections accessed through the GBIF data portal such as Botanic Garden and Botanical Museum Berlin-Dahlem, European Environment Agency, Fairchild Tropical Botanic Garden, Real Jardin Botanico Madrid, Vascular Plant Herbarium, International Botanical Collections GBIF-Sweden, Herbarium Hamburgense, Herbarium of the University of Aarhus, Museum National d'Histoire Naturelle, National Herbarium of New South Wales, Royal Museum of Central Africa and University of Hohenheim. In addition, we also obtained data through literature surveys as well as from private databases and observations held by field botanists (see acknowledgements). For very conspicuous species (*Elaeis guineensis, Hyphaene petersiana, Phoenix reclinata*) we used Google Earth satellite imagery to achieve more palm localities[1,2]. We have accessed the following websites to retrieve information on palm occurrences: www.zimbabweflora.co.zw (last accessed March 2009); www.aluka.org (last accessed December 2007); www.unesco.org (last accessed December 2007); www.iucnredlist.org (last accessed December 2007).

A potential problematic issue for the use of primary occurrence data is taxonomy. As taxonomy is a dynamic field of research, nomenclature is continuously updated. For the African palm database it has been a primary goal to identify taxonomic issues arising from misidentifications or outdated taxonomy. Hence, the database has been meticulously scrutinized for any taxonomic issues by: 1) following the nomenclature of the most recent revisions of African palm taxa, 2) identifying misidentifications by comparing duplicate herbarium records from multiple herbaria, 3) plotting all records in geographical space and identifying geographical outliers, with attention to inconsistencies between their known distribution and the mapped point localities. All issues have been addressed under supervision by Dr. John Dransfield, Royal Botanic Gardens, Kew, a world-leading specialist on the palms of Africa (and in many other regions).

Additionally, the point localities were verified in geographical space to identify erroneously georeferenced specimens. Locality descriptions (e.g., country) of herbarium specimens were checked against an underlying map of political boundaries in Africa and errors such as switched latitude and longitude and in negative and positive decimal degree values were identified and corrected. We subsequently extracted occurrence records for 40 palm species (representing 82.5% of all recognised African palm species) with ≥ 10 unique occurrences at 10 × 10-km resolution which covered in total 2066 unique occurrence records (mean: 51.7; median: 39) (Supplementary Table S1). To accommodate the uncertainty in some georeferences (7-10 km), we chose to set the analytical unit of the study at a resolution of 10 × 10-km. The occurrence records were then spatially thinned retaining only one occurrence record per 10 × 10-km equal-area grid cell.

Due to different climatic affinities between groups of African palms we divided the palms into two distinct groups based on ecology[3], namely rainforest and open-habitat palms. The rainforest group consisted of strictly rainforest palms and the open-habitat group of desert, grassland and savanna palms tolerant of more exposed habitats and never found in forests[4]. Two species are not restricted to rainforest or open habitats (*Elaeis guineensis* and *Phoenix reclinata*) and where not included in any category, but counted towards the total palm species richness estimates.

Climate data.

Climatic data was obtained for the baseline period (1960−1990) from the Worldclim database[5]. Future climate data was obtained for 2010−2039 (referred to as 2020), 2040−2069 (2050) and 2070−2099 (2080) from the International Centre for Tropical Agriculture (CIAT) dataset of the Fourth Intergovernmental Panel on Climate Change (IPCC) report using the spatial downscaling data of the Delta Method[6]. To make sure the models covered a wide range of future climatic scenarios, future climatic data layers were derived from three different coupled Atmosphere-Ocean global circulation models (GCMs) and three future greenhouse gas emission scenarios (SRES)[7]. The SRES scenarios represent a range of underlying driving forces (demographic, economic and technological) of greenhouse gas and sulphur emissions. They are developed based on a wide range of assumptions regarding these driving forces to represent the uncertainty of the future. We selected the scenarios B2a, A2a and A1b as they represent a variety of assumptions regarding the

driving forces and thus encompass a range of potential future demographic, social, economic, technological and environmental developments. The A1b scenario is one group within the A1 family, which characterizes a future world where the human population continues to grow until it peaks in ca. 2050 and afterwards declines. Economic growth is rapid and technological development is fast and innovative. The A1b group differs from other A1 groups in the direction of change in the energy system which for A1b is assumed to be a balanced reliance on all sources of energy (non-fossil energy to fossil intensive). The A2a represents the A2 family which describes a future heterogeneous world where the human population continues to grow to towards 2100. The economy develops regionally and technological change is slow compared to other scenarios. Finally B2a of the B2 family of scenarios is one of the less severe scenarios, where the human population continues to grow, but at a lower rate than the A2a scenario. Focus is more on local solutions to secure environmental sustainability and social equity[7].

At the time of data download (January 2012), climate layers for the combination of the three SRES and three time periods were not available for all GCMs. Hence, out of the available GCMs, we selected data derived from three GCMs, namely CCCMA-CGCM2 (referred to as CCCMA), CSIRO-MK2 (CSIRO) and HACCPR-HADCM3 (HADCM), with different original resolutions of 1.9º x 1.9º, 0.8º x 1.9º and 1.25º x 1.25º with climate sensitivities ranging between 3.1º C-3.4º C[6]. We further mapped the distribution of absolute differences in annual mean temperature and water balance between pairs of GCMs to make sure they all had marked spatial variation in the climatic values which would transcend into spatial variation in predicted suitability. The three chosen GCMs showed clear spatial variation across Africa with absolute differences ranging between 0ºC-3.5ºC for annual mean temperature and between 0 mm-6620 mm for annual water balance across Africa for the 2080 scenarios (Supplementary Fig. S8-S9). For A1B the above GCMs were not available at the time of download and we therefore used CCCMA-CGCM3 for CCCMA, CSIRO-MK3.1 for CSIRO and UKMO-HADCM3 for HADCM, respectively.

For the baseline and each of the combinations of GCMs and SRES for all three future time intervals we obtained three temperature variables (annual mean temperature, minimum temperature of the coldest month and temperature seasonality), as well as monthly data on precipitation and temperature. All

datasets were originally in 5' resolution, but were reprojected to the Lambert Azimuthal Equal Area (LAEA) projection using bilinear interpolation at 10 × 10-km resolution in ArcGIS.

Computation of potential evapotranspiration (PET) and water balance (WATBAL).

One of the challenges to correctly simulate future distributions of plants in species distribution modelling (SDM) is to account for the effect of CO_2 fertilization under future increased CO_2 concentrations[8]. This fertilization potentially changes plant water use efficiency by decreasing plant transpiration and evapotranspiration[9]. Failing to account for the CO_2 effect in SDM could potentially make modelling outputs unreliable. A solution to account for such effects is to rescale climate predictors to better represent how they are functionally perceived by plants under given CO_2 concentrations[8]. For the present study we refrain from using purely precipitation-derived variables. Instead, we constructed water balance-derived variables as these are based on the monthly differences between precipitation and PET, because it has recently become possible to rescale PET to take into account the potential CO_2 effect on plants[9].

Kruijt *et al.*[9] has generated rescaling factors (c-factors) for PET across four different vegetation and plant functional types under two different CO_2 concentration rises relative to the present-day level. We used the mean c-factors of Kruijt *et al.*[9] of all four categories (Table S4) and fitted a linear regression for each category between c-factors and the CO_2 concentration rises making the assumption that if the CO_2 concentration is unchanged (0) the resulting c-factor would be 1 meaning that PET is unchanged (Fig. S7). The resulting regression equations were subsequently used to extrapolate the c-factors to different future CO_2 concentration rises. Future CO_2 concentration rises were computed as the difference between future and baseline CO_2 concentrations. Baseline CO_2 was averaged over the years 1970−1990 (only available from 1970) to be more consistent with present-day climate from the Worldclim dataset. The projected future concentrations were obtained for three time periods 2020 (average of the years 2010−2040); 2050 (average of the years 2040−2070) and 2080 (average of years 2070−2100) to be consistent with the future climate data. The future CO_2 concentrations were derived for the individual SRES (A1B, A2A, B2A) from the Bern and ISAM models of the Intergovernmental Panel on Climate Change[10]. We averaged the resulting c-factors for the four habitat types (C1−C4) for each CO_2 concentration rise and subsequently also

averaged that resulting c-factors for the Bern and ISAM models per SRES per year interval (Table S5). These nine final c-factors were subsequently used to rescale PET.

We used the monthly temperature data for the baseline and the future time periods to compute PET of each month using the method of Thornthwaite[11]. The Thornthwaite-type PET is purely temperature based and given by: $PET = 1.6 *(10 * T / I)^a$, where T is the mean monthly temperature, I is the heat index for a given area computed as the sum of values *i* calculated for the 12 months of the year given by: $i = (T/5)^{1.514}$ and $a = 0.000000675 * I^3 – 0.0000771* I^2 + 0.01792 * I + 0.49239$. The above formula computes PET based on a standard month of 30 days and on days with 12 hours sunlight. Since this varies with month and latitude the monthly PET values were adjusted for day and month length using the correction factors given by Thornthwaite[11] across latitudes spanning Africa.

We constructed three water balance variables namely annual water balance (WATBAL; mm/year) computed as the annual sum of the monthly differences between precipitation and PET, water balance seasonality computed as the standard deviation of monthly water balance, and water balance of the driest quarter computed as the minimum sum of water balance across three consecutive months. To account for the potential effect of CO_2 fertilization under increased CO_2 concentrations, we created a second set of the water balance-derived variables using the rescaled PET based on the CO_2 concentrations of the different gas emission scenarios (A1B, A2A, B2A) as explained above (Fig S7, Table S4-S5).

Species distribution modelling

We used two advanced machine-learning modelling techniques to fit species distribution models for the 40 palm species, namely Maximum Entropy (Maxent) species distribution modelling, run in Maxent v. 3.3.3k[12] and Generalised Boosting Model (GBM)[13] modelling, run in R[14] using BIOMOD v. 1.1.7[15]. Maxent and GBM are commonly used SDM algorithms[16,17] and in comparative studies they have been found to be among the best performing SDM techniques[18,19]. We have previously evaluated different methods (Maxent, GBM, GLM, and GAM) for their ability to predict the current ranges of the African palms based on evaluation statistics and by expert knowledge. We found GBM and Maxent to provide the overall best results[3], and therefore they were chosen for the current study.

We created two distribution models per species. First, we used the baseline climatic data (temperature and water balance-derived variables) combined with six spatial filters to predict present-day distributions (SPATIAL model). We used spatial filters to take into account any potential spatial constraints on the species' actual distributions[20]. We selected six spatial filters to equal the number of climate variables as this has previously been found useful for predicting African palm distributions[21]. Spatial filters are orthogonal variables (eigenvectors) representing the spatial relationship amongst spatial units derived from a Principal Coordinate Analysis based on a truncated pairwise distance matrix of the geographic position of each spatial unit at various scales from broad to fine-scale spatial patterns[22]. Spatial filters were computed in SAM 3.0[23] using default settings, further geoprocessed as described in Blach-Overgaard *et al.*[21], and resampled to 10 × 10 km resolution, for the current study. Secondly, we constructed an additional model using just the six climatic variables only (CLIMATE model) to produce a potential climatic model for the baseline. The CLIMATE model was subsequently projected onto two different sets of future climate data for all combinations of GCMs and SRES for the three time periods. The first dataset consisted of three temperature and three un-scaled water balance variables (−CO_2), while the second set was the same set of temperature variables combined with three CO_2-modified water balance variables (+CO_2).

For the GBM, we chose default settings of BIOMOD running maximum 3000 trees and five cross validations to select the optimal number of trees. Finally, for Maxent we also chose default settings as they have been shown to provide overall robust results[24]. For all models, we used 10,000 randomly selected pseudo-absences as a random selection of pseudo-absences has been shown to outperform other selection techniques[25]. Model evaluation of both models was done by calibrating the baseline models on 80% of a random sample of the initial data and evaluated against the remaining 20% using the areas under the receiver operating curve (AUC)[26] and the true skill statistic (TSS)[27]. Model evaluation was performed 10 times, each time selecting a different 80% random sample and evaluating model accuracy against the remaining 20%. We averaged the AUC and TSS for each model-run of the SPATIAL and CLIMATE models using the GBM and Maxent algorithms. Models with AUC values ≥ 0.75 are considered to produce good reliable predictions[28]. TSS ranges from -1 to +1, where +1 indicates a perfect model fit and values ≤ 0 indicates models which are no better than random[27]. More specifically, we interpreted the following ranges: 0.2−0.4 as

poor; 0.4−0.6 as fair; 0.6−0.8 as good; and 0.8−1.0 as excellent. In general, all palm species distributions modelled by Maxent and GBM were statistically well-predicted, with the majority of models having AUC > 0.90 and TSS > 0.75 with a few exceptions (Table S6). For *M. argun* the SPATIAL model was poorly predicted by GBM as interpreted from the AUC and TSS statistics (Table S6), but also verified visually. Hence further processing of the output from the SPATIAL model relied solely on the Maxent predictions for *M. argun* to avoid any inconsistencies. For the final calibration of each model used for making predictions and projections we used 100% of the available data to avoid uncertainties in models by random removal of presences[29].

We chose to create two related models (based on the same climatic predictors) per species to be able to estimate the change in pure climatic suitability given by the CLIMATE model between the baseline and the three future time periods within realistic present-day range margins and within the margins of potential colonized areas of the species (deducted from the SPATIAL model). The SPATIAL model combined with a statistically set threshold produced an estimation of a given species' actual distribution as the spatial filters constrain the model to reduce overpredictions into climatically suitable areas at far distances from any known occurrence records (Fig. S10). Processing of the outputs of each model type is explained in further detail below.

Construction of dispersal scenarios

We created two dispersal scenarios for the present study, namely a no-dispersal scenario where palms do not disperse outside their predicted current range limits and a 100-km-dispersal scenario where palms can disperse up to 100 km from their current range limits in all directions. The 100-km-dispersal scenario is a more realistic and conservative migration limit in relation to tropical trees[30] relative to the unrealistic full migration scenario[31] often applied in species distribution modelling studies[32,33]. To construct current actual distributions for all species we converted the continuous suitability prediction of the SPATIAL model to binary predictions using the threshold equal training sensitivity and specificity in Maxent. This corresponds to the optimized ROC threshold of BIOMOD, which minimises the absolute difference between sensitivity and specificity and is ranked among the most reliable thresholds[34]. We subsequently extracted the combined

area predicted as presence by Maxent and GBM except for *M. argun* (see above). To obtain a range as close to each species actual distribution each species' distribution was examined for extreme outliers, grids predicted as presences, but occurring at far distances from any coherent group of presence grids and outside the known distribution. For most species this was mostly cells at a distance of more than 1000 km and for only seven species below 1000 km (between 140−700km distances). These were removed from the predictions and the resulting outputs were used as the species' no-dispersal masks. To create the 100-km-dispersal mask we extended the no-dispersal mask with 100 km in all directions (Fig. S10).

Processing the modelling results.

We refrained from converting the future continuous suitability projections of the Maxent and GBM CLIMATE models to binary predictions to avoid information loss and the uncertainties associated with the choice of thresholds[35]. Instead, we made all further processing of the modelling outputs based on the suitability scores of the baseline CLIMATE model and the 4320 future projections for 40 species across each possible modelling combinations (two SDMs × three GCMs × three SRES × three time periods × two CO_2-scenarios) within the species-specific no-dispersal or 100-km-dispersal masks and disregarded the suitability scores outside the masks (Fig S10). For GBM all model outputs are by default ranging from 0−1000 and as all further processing relies on suitability scores ranging between 0−1, we divided all GBM outputs by 1000.

Assessments of the impact of climate change.

We assessed the impact of climate change by first calculating the change in climatic suitability as the difference in climatic suitability between baseline (CLIMATE model) and future conditions following Hof *et al.*[36] (Fig. S10). This was done for all 40 species across all model combinations (SDM × GCM × SRES) for the three time periods and for both CO_2 scenarios within both dispersal masks. We identified the regions with the highest projected impact of climate change on palm richness patterns (all palms, rainforest palms and open-habitat palms) within the no-dispersal mask by summing the number of species per 10 × 10-km grid that (1) lose climatic suitability, termed 'local climate losers' (negative change in climatic suitability between future and current conditions), (2) gain climatic suitability, 'local climate winners' (positive change in

climatic suitability between future and current) and (3) show no change. Species were considered 'losers' or 'winners' regardless of the magnitude of the change in climatic suitability and could be counted as winners in one grid cell and losers in another grid cell. To estimate the climate change effects on immigrant species we summed the number of species per 10 × 10-km grids which gained climatic suitability outside the no-dispersal mask, but within the 100-km-dispersal mask, termed 'immigrant winners' (positive change in climatic suitability between current and future conditions). We further calculated the proportion of 'local climate losers' and 'local climate winners' of the total species pool per grid cell.

To determine the overall loss in climate suitability per species we summed the number of grid cells (10 × 10 km) projected to lose climate suitability within the no-dispersal mask and subtracted the number of grid cells projected to gain climate suitability in the band between the margins of the no-dispersal and 100-km-dispersal masks. Proportion of overall loss of climate suitability was computed by dividing this difference by the total number of grid cells in the no-dispersal mask. To estimate the effect of the potential water use efficiency under increased CO_2 we calculated the percentage difference in proportion overall loss of climate suitability between the two CO_2 scenarios. This was done for all combinations of SDM × GCM × SRES for all time periods. A final consensus' was made as an arithmetic mean across SDM × GCM × SRES per time period.

Consensus maps were derived by calculating arithmetic means of the richness and proportions of 'local climate losers', 'local climate winners' and 'immigrant climate winners' across all model combinations (SDM × GCM × SRES) for the three time periods and two CO_2 scenarios. As it is well-known that there is large differences in modelling output depending on the given SDM, GCM or SRES used[37] we further mapped the 'local climate loser' richness patterns (all palms, rainforest palms and open-habitat palms) for 2080 for the +CO_2-scenario separately as arithmetic means for (1) all combinations of SDM × GCM per SRES (Fig. S4), (2) across all combinations of SDM × SRES per GCMs (Fig. S5) and finally (3) across all combinations of GCMs × SRES per SDM (Fig. S6), to assess the uncertainty around the consensus.

Human population density scenarios

To estimate the potential future threat from humans on palms we constructed human population density projections for three time periods centred on 2020, 2050 and 2080. We used the Gridded Population Density map of the World in 2.5' resolution for year 2000[38]. The global map was reprojected to the LAEA projection and resampled to 10 × 10 km resolution extracted only for continental Africa. We modified the 2000 layer using the latest population projections on human population densities (people/km^2) by the United Nations (http://esa.un.org/unpd/wpp/unpp/panel_population.htm) which incorporates major influences on demographic trends available at country level. We obtained data on these country-level human population density projections for year 2000 to 2100 in 5-year intervals for all countries across continental Africa. These were subsequently averaged across three 30-year intervals to match the future climate data, 2010−2040 (2020), 2040−2070 (2050) and 2070−2100 (2080). The per cent increase in human population densities (HPD) from 2000 to the three different future time periods (2020, 2050 and 2080) were computed per country in Africa. We multiplied country-based percentage HPD increases for 2020, 2050 and 2080 in a cell-by-cell manner to the 2000 gridded population map thereby constructing three future human density maps (HPD2020, HPD2050 and HPD2080; Fig. S11).

Assessment of the impact of human population density.

We assessed the human population density change over time for each species by weighting the time-specific human population density maps (HPD2020, HPD2050 and HPD2080) by the proportion of suitability per grid within each dispersal mask given by the species specific distribution models for all combinations of models (SDM × GCM × SRES × CO_2-scenario) matched with the specific time period (e.g., HPD2020 with SDM × GCM × SRES × CO_2-scenario for year 2020). The proportion of suitability was computed for each grid by dividing the suitability of each grid within the species specific dispersal masks by the total sum of suitability per species across all grids within the given dispersal mask. The human population density per species per time period was subsequently calculated by summing up the weighted HPD values across all cells within either the no-dispersal or 100-km-dispersal masks. We obtained a consensus averaging by

calculating the arithmetic mean across all model combinations (SDM × GCM × SRES) per time slice and CO_2 scenario.

To assess whether palms species, by the end of the century, are likely to occur under HPD levels, which potentially could lead to increased risk of habitat loss and overexploitation, we set a threshold for high HPD corresponding to the continental-wide average HPD for year 2000 (CON-HPD; 50.14 people/km2; Table S2). To assess how HPD is related to impacts on palm populations, we assessed the link between HPD and land cover transformation, one of the most important drivers of extinction risks in palms[39], computing summary statistics of HPD for year 2000 within each land cover class in Africa given by the Land-cover map of Africa produced as a part of the Global Land Cover project in 2000 at 1 × 1-km resolution[40]. We resampled the Gridded Population Density map of the World for year 2000[38] to 1 × 1-km resolution and extracted the mean, median and standard deviation of HPD within each land cover type using Zonal statistics in ArcGIS and listed these in relation to the continental-wide average HPD for year 2000 (CON-HPD; 50.14 people/km2; Table S2). This analysis showed that most natural land cover classes have mean HPD below CON-HPD, while most anthropogenic land cover classes and especially those in which most palm species would not be able to maintain populations (e.g., irrigated croplands and croplands >50%) have HPD above the CON-HPD threshold (Table S2).

Protected area network.

To assess the future role of the conservation network in Africa for African palms, we used the World database of Protected Areas[41] from 2009. The protected area network contains points and polygon data for protected areas and these were dissolved and the layer was subsequently clipped and reprojected to match the climatic data. For each 10 ×10 km grid cell across Africa we obtained the proportion of the cell covered by conservation areas (Fig. S12).

Assessment of the impact of climate change on conservation areas.

To assess the number of species gaining or losing climate suitability within the existing protected area network in Africa in the future we applied an index derived from a probabilistic estimation of the matching

between species' climate suitability and the proportion of grid cells that is conserved following the approach of Araújo *et al.*[42]. The matching of climatic suitability with conserved area (conserved suitability; CS) was done by multiplying the proportion of grid cell *n* covered by protected area with the suitability score of grid cell *n* estimated by species-specific distribution models (CS*n*) on a cell-by-cell basis. Both measures range from 0 to1, so resulting values for CS range from 0 for grids which are unsuitable for a given species or with no areas conserved, to 1 for cells with climate suitability equal 1 which are fully conserved. For each combination of models (SDM × GCM × SRES × CO_2-scenario) for each time period (baseline, 2020, 2050 and 2080) we quantified the expected climate suitability for each species within protected areas (CS) as the sum of CS*n* within the species specific no-dispersal and 100-km-dispersal masks. Changes in CS were calculated for each species within each of the dispersal masks between CS of the baseline period and each of the future time periods. Species projected to have increased climate suitability in protected areas in Africa were termed 'conservation winners' (CSFuture/CSBaseline > 1), while species projected to have decreased climate suitability in protected areas in Africa were termed 'conservation losers' (CSFuture/CSBaseline < 1). We assessed the number and proportion of all palms, rainforest palms and open-habitat palms projected as winners and losers by summing the number of winner and loser species per group and dividing the sum by the total number of palm species per group. Consensus results were obtained by calculating arithmetic means across all model combinations (SDM × GCM × SRES) for each time period and CO_2-scenario.
All GIS analyses were done using Python 2.5 and ArcGIS 9.3 (ESRI, Redlands, CA, USA).

Supplementary figures

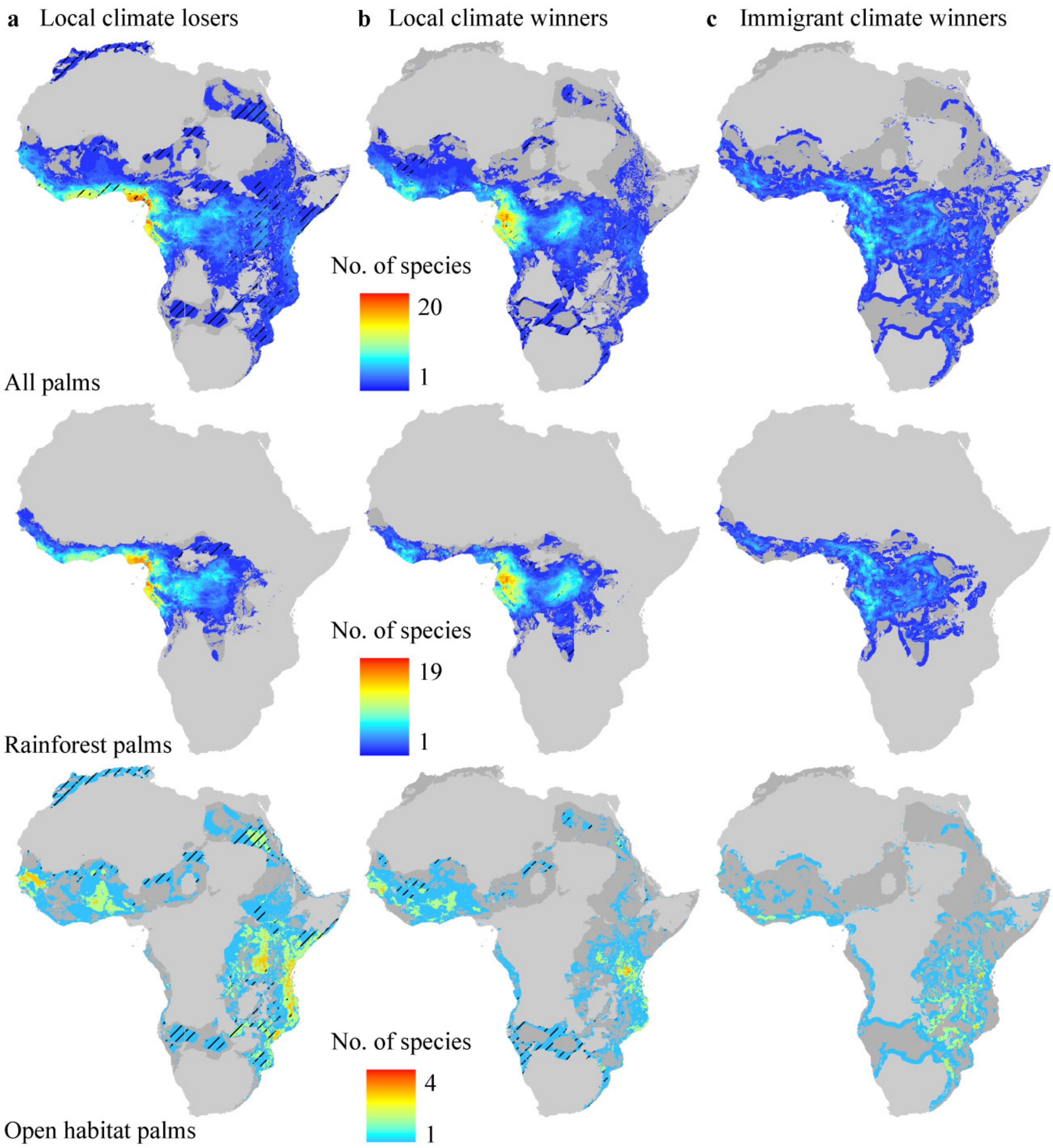

Figure S1 | Three measures ('local climate losers', 'local climate winners' and 'immigrant climate winners') of the projected impact of climate change for year 2020. **a**, Spatial overlap of the number of species per grid and the highest proportion of species (80−100%) projected to lose climate suitability in a given area. **b**, Spatial overlap of the number of species per grid and the highest proportion of species (80−100%) projected to gain climate suitability in a given area. **c**, Number of species per grid projected to gain climate suitability outside their current range at distances up to 100 km in a given area. Values for **a-c** computed as arithmetic means across two species distribution models, three global circulation models and three gas emission scenarios for the +CO_2-scenario (Supplementary Methods). The light grey areas in all panels indicate the absence of palms, while the darker shade grey denotes the predicted baseline range limit of a particular palm group. The black hatched areas in **a** and **b** indicate proportions (80−100%) of climate

losers or winners of the total species pool in a given area. The map was created in ArcGIS 9.3 (ESRI, Redlands, CA, USA).

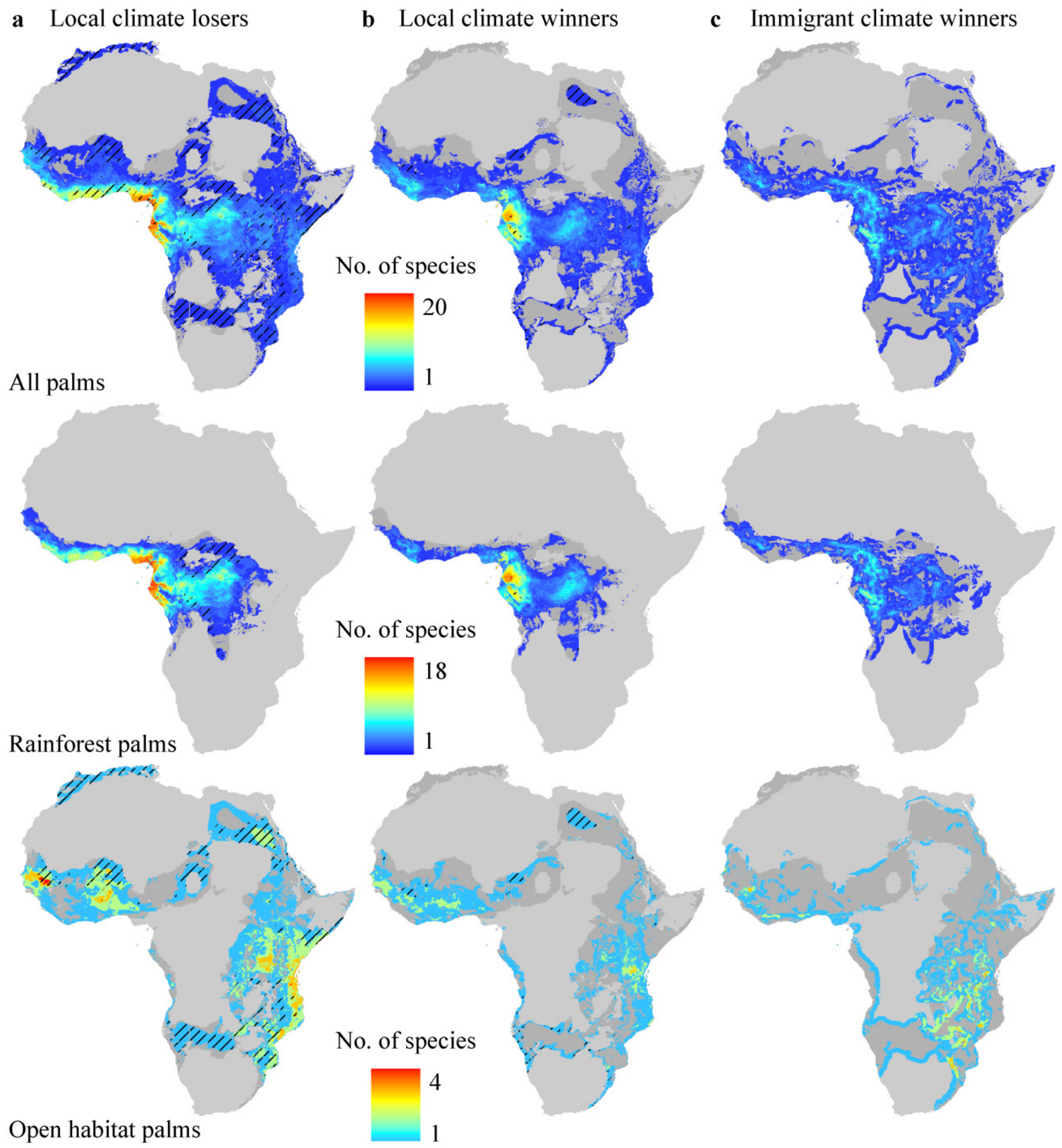

Figure S2 | Three measures ('local climate losers', 'local climate winners' and 'immigrant climate winners') of the projected impact of climate change for year 2050. **a**, Spatial overlap of the number of species per grid and the highest proportion of species (80−100%) projected to lose climate suitability in a given area. **b**, Spatial overlap of the number of species per grid and the highest proportion of species (80−100%) projected to gain climate suitability in a given area. **c**, Number of species per grid projected to gain climate suitability outside their current range at distances up to 100 km in a given area. Values for **a-c** computed as arithmetic means across two species distribution models, three global circulation models and three gas emission scenarios for the +CO_2-scenario (Supplementary Methods). The light grey areas in all panels indicate the absence of palms, while the darker shade grey denotes the predicted baseline range limit of a particular palm group. The black hatched areas in **a** and **b** indicate proportions (80−100%) of climate losers or winners of the total species pool in a given area. The map was created in ArcGIS 9.3 (ESRI, Redlands, CA, USA).

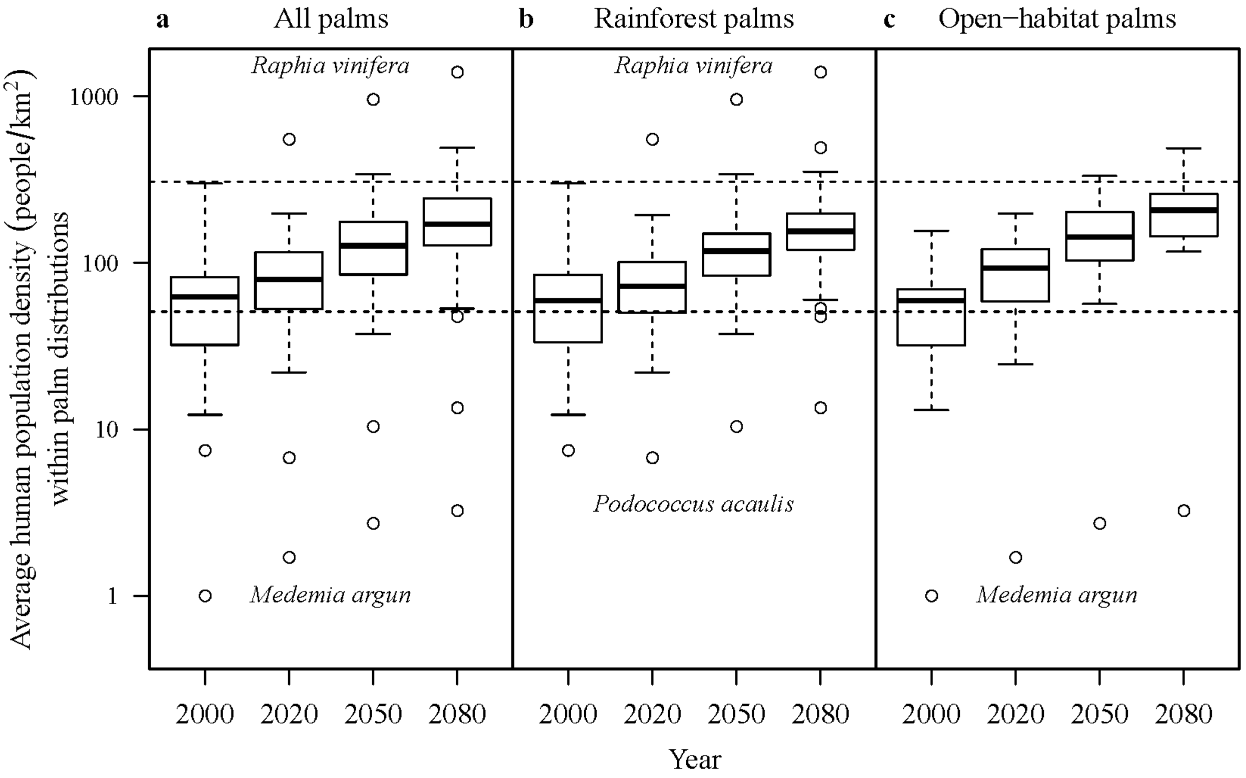

Figure S3 | Average human population density within areas of palm distributions. Average human population density (on log_{10} scale) within no-dispersal ranges of **a,** all palms (*n* = 40), **b**, rainforest palms (*n* = 27) and **c**, open-habitat palm (*n* = 11) palms for the +CO_2 scenario (Supplementary Methods) for four time periods in the 21st century. The upper and lower dotted lines mark population density in the densest populated country in Africa in year 2000 (Rwanda; 307 people/km^2) and the continental-wide average human population density in year 2000 (50.14 people/km^2), respectively. The most extreme outlier species for each group and time slice are named. Values computed as arithmetic means of all model combinations of two species distribution models, three global circulation models and three gas emissions. The horizontal black bar is the median for each group per time period. The box indicates the interquartile range and whiskers extend to the most extreme data point, which is no more than 1.5 times the interquartile range from the box, while more extreme values are displayed as open circles.

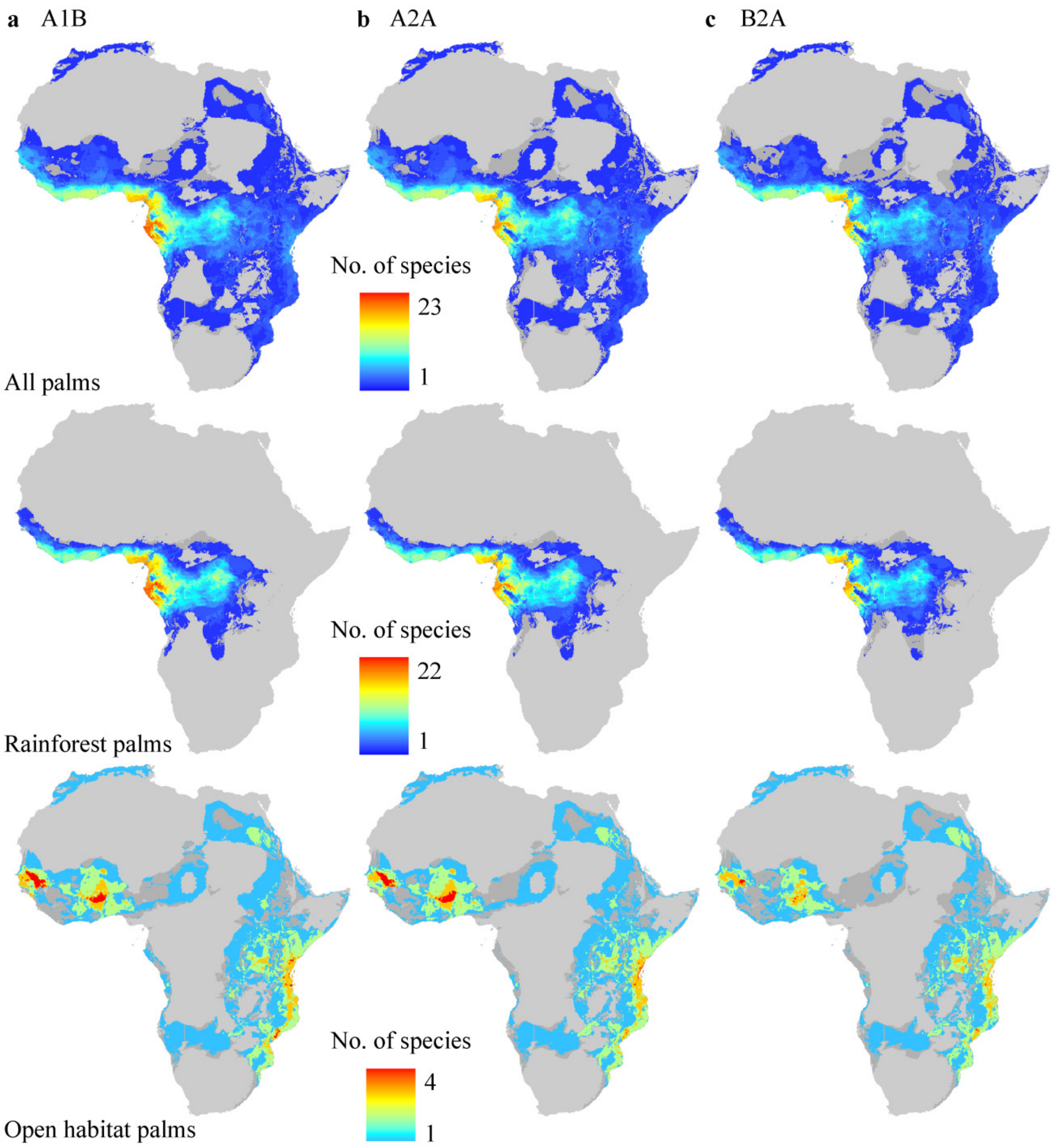

Figure S4 | Geographic variation in the number of 'local climate losers' for different gas emission scenarios, projected for the year 2080 for the +CO_2 scenario. **a,** A1B; **b**, A2A and **c**, B2A for all, rainforest and open-habitat palms. Values are computed as arithmetic means across two species distribution models and three global circulation models. The light grey areas in all panels indicate the absence of palms, while the darker shade grey denotes the baseline range limit of a particular palm group. The map was created in ArcGIS 9.3 (ESRI, Redlands, CA, USA).

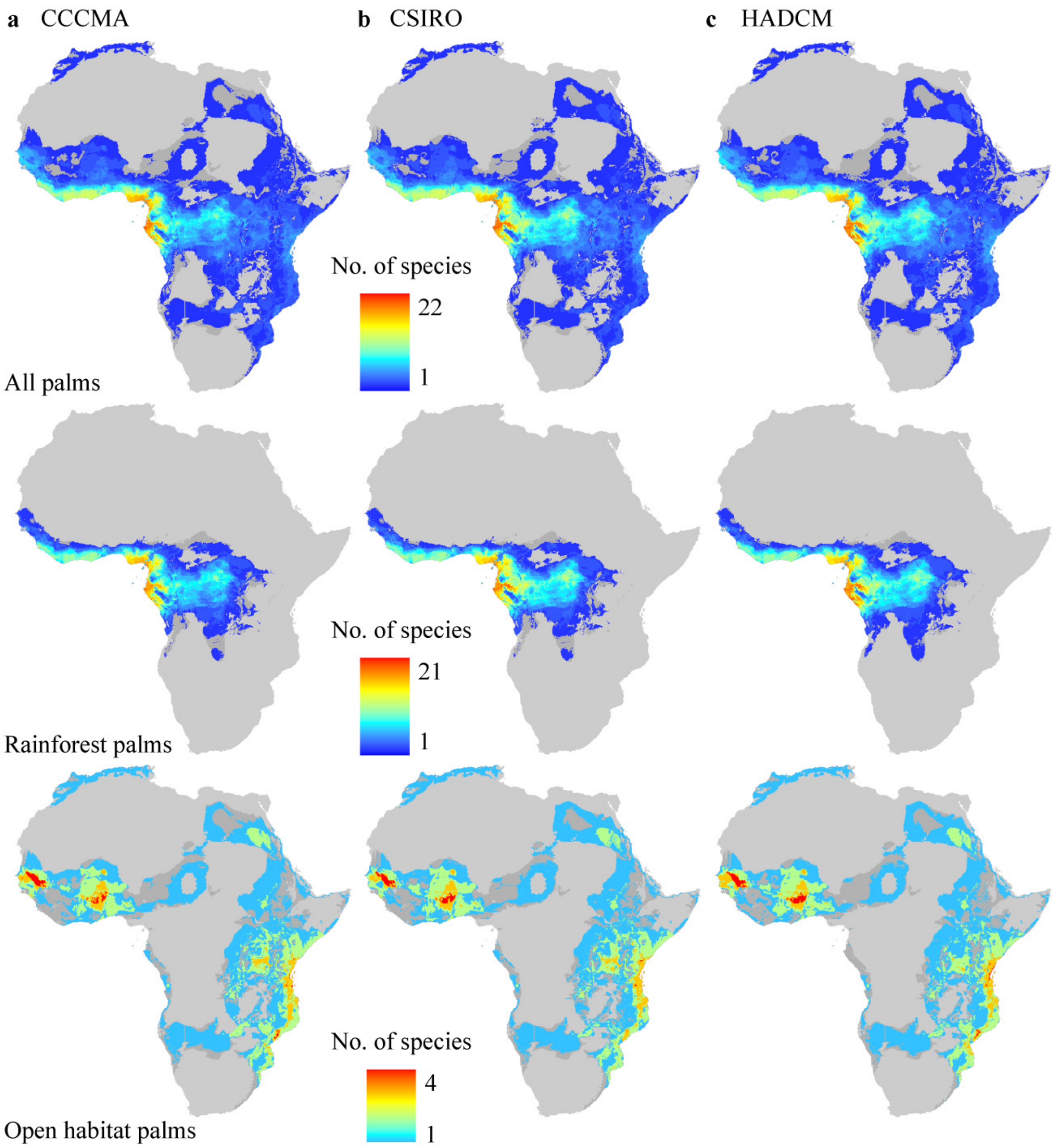

Figure S5 | Geographic variation in the number of 'local climate losers' for different global circulation models, projected for the year 2080 for the +CO_2 scenario. **a,** CCCMA; **b**, CSIRO and **c**, HADCM for all, rainforest and open-habitat palms. Values are computed as arithmetic means across two species distribution models and three gas emissions. The light grey areas in all panels indicate the absence of palms, while the darker shade grey denotes the baseline range limit of a particular palm group. The map was created in ArcGIS 9.3 (ESRI, Redlands, CA, USA).

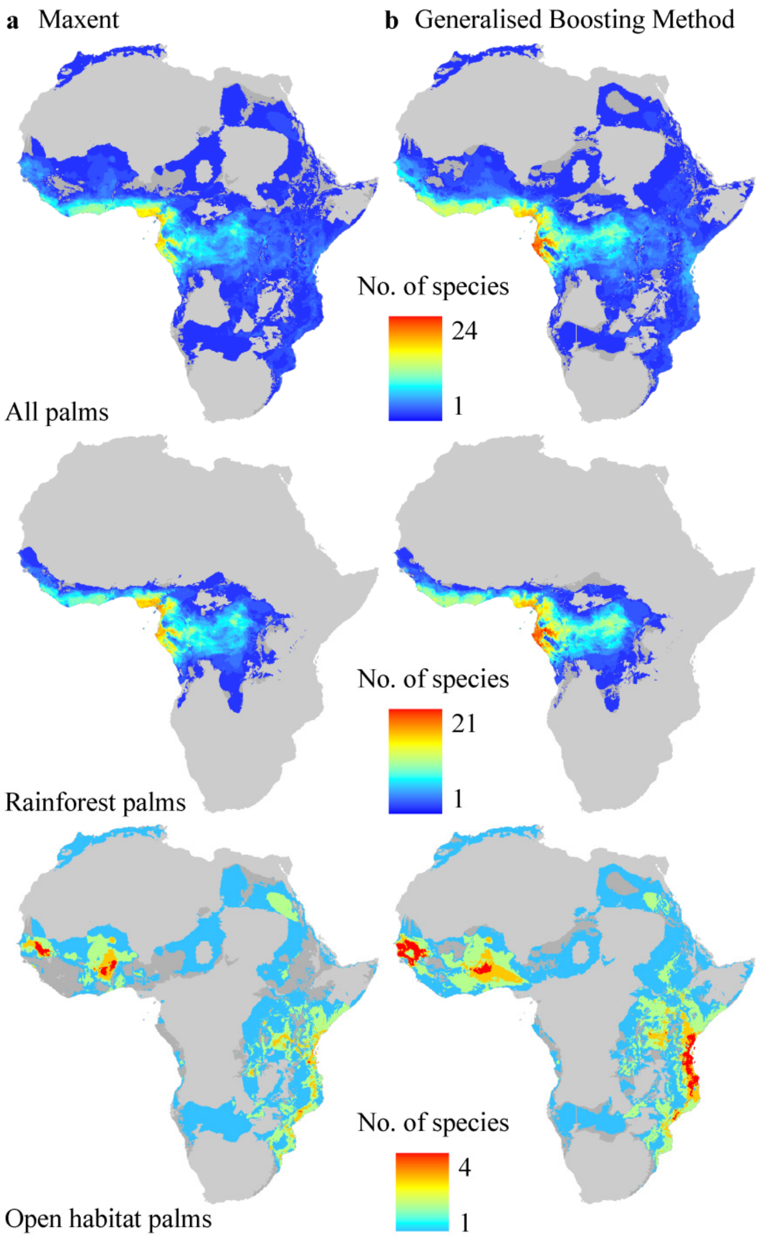

Figure S6 | Geographic variation in the number of 'local climate losers' for different species distribution models, projected for the year 2080 for the +CO_2 scenario. **a,** Maxent and **b**, Generalised Boosting Method for all, rainforest and open-habitat palms. Values are computed as arithmetic means across three global circulation models and three gas emissions. The light grey areas in all panels indicate the absence of palms, while the darker shade grey denotes the baseline range limit of a particular palm group. The map was created in ArcGIS 9.3 (ESRI, Redlands, CA, USA).

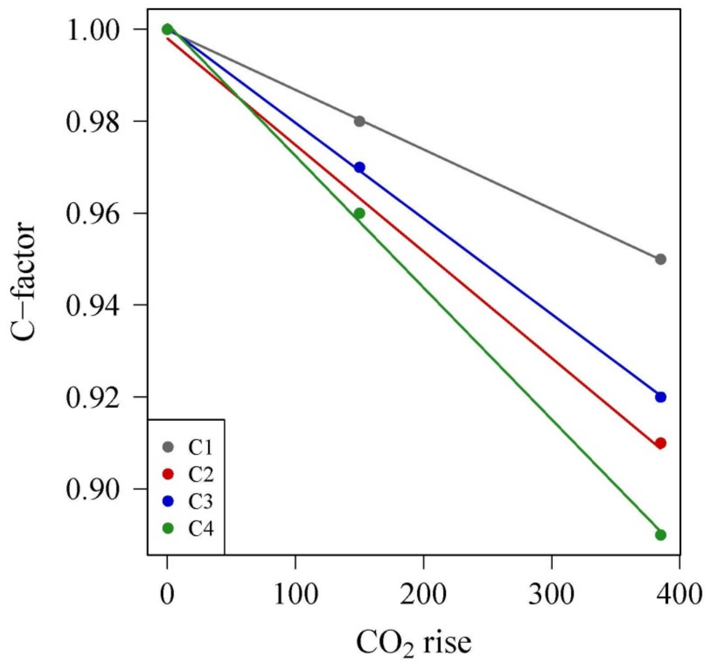

Figure S7 | Fitted linear regressions of four (C1, C2, C3, C4) correction factors (c-factor) for potential evapotranspiration as a function of CO_2 rise. The c-factors are given by Kruijt *et al.*[9]. See supplementary Table S4 for specific values.

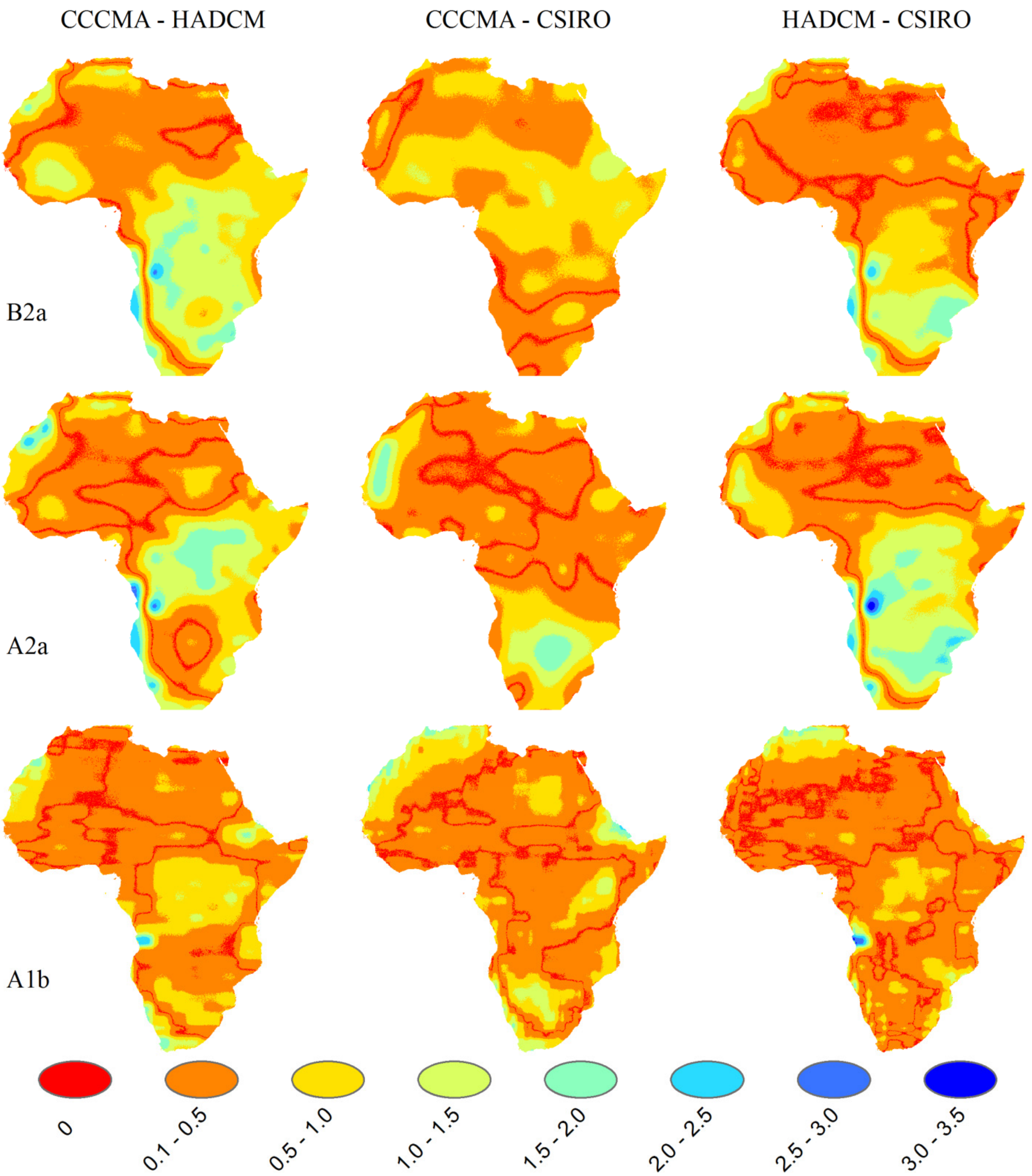

Figure S8 | Geographic variation of absolute differences in annual mean temperature (ºC) between pairs of Global Circulation Models (GCMs) at 2080. The vertical panels show the absolute difference in ºC of the variable annual mean temperature between two GCMs (CCCMA versus HADCM; CCCMA versus CSIRO; HADCM versus CSIRO) for three different gas emission scenarios (B2a, A2a, and A1b). The map was created in ArcGIS 10.2.2 (ESRI, Redlands, CA, USA).

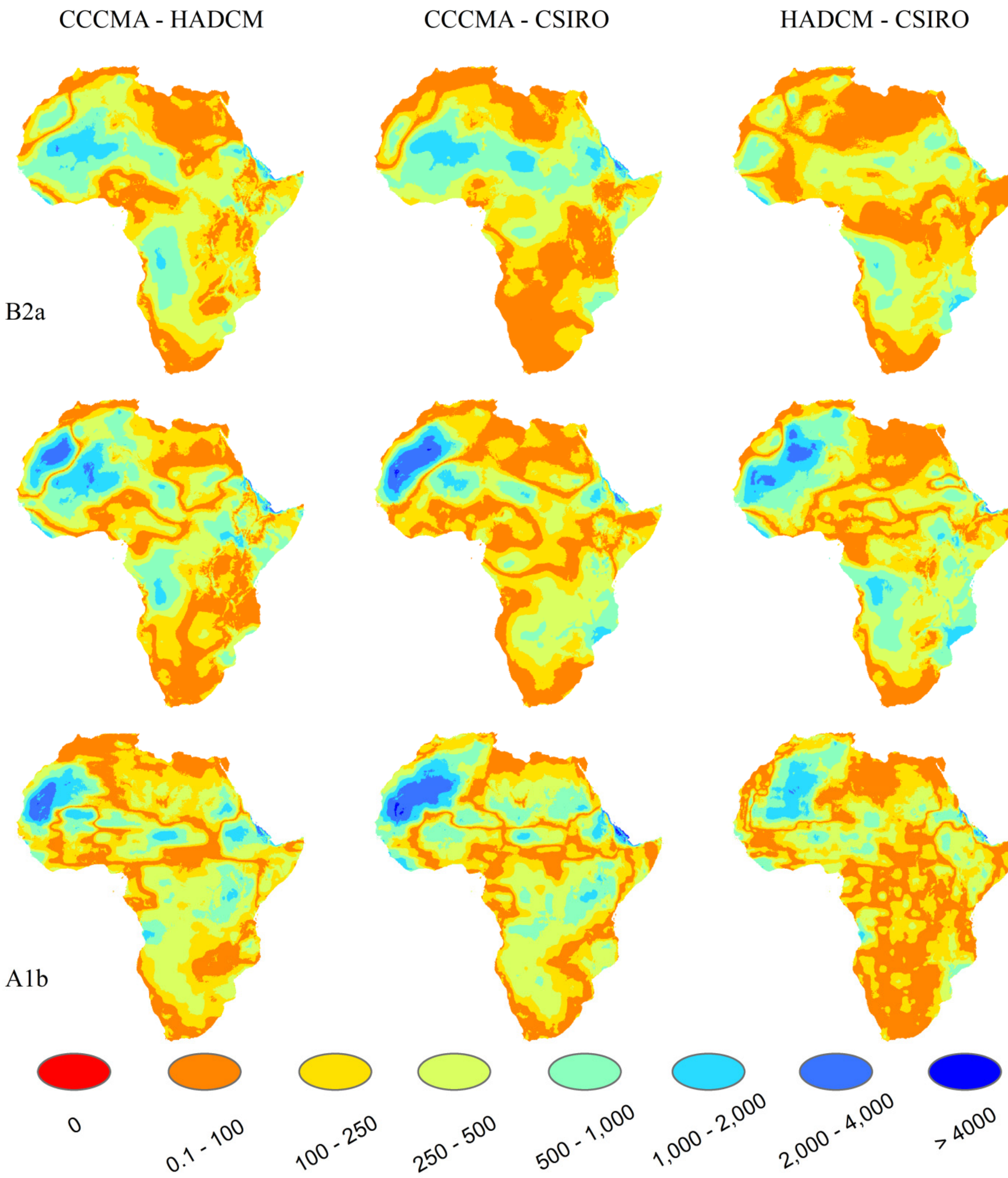

Figure S9 | Geographic variation of absolute differences in annual water balance (mm) between pairs of Global Circulation Models (GCMs) at 2080. The vertical panels show the absolute difference in mm of the variable annual water balance between two GCMs (CCCMA versus HADCM; CCCMA versus CSIRO; HADCM versus CSIRO) for three different gas emission scenarios (B2a, A2a, and A1b). The map was created in ArcGIS 10.2.2 (ESRI, Redlands, CA, USA).

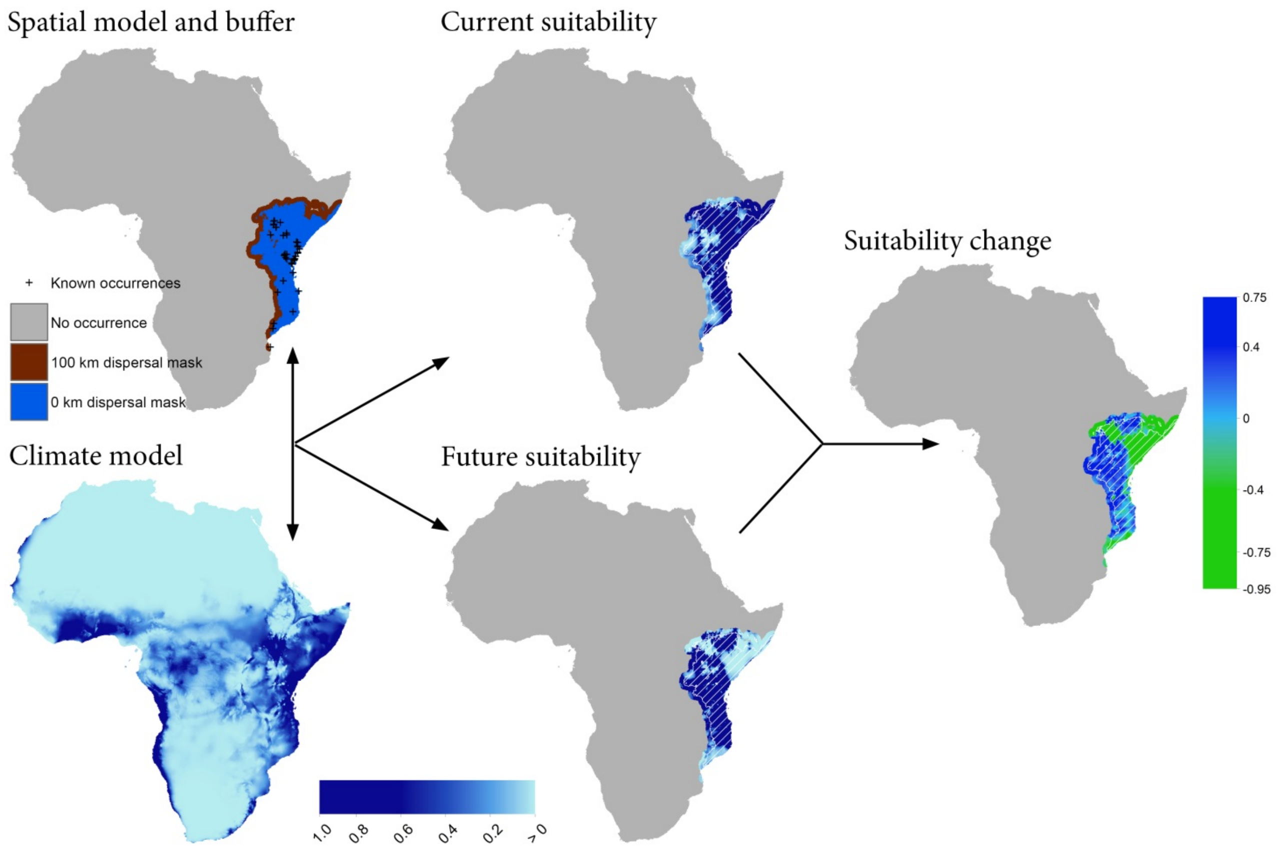

Figure S10 | Flow diagram of the steps in estimating the change in climatic suitability between time periods for two dispersal scenarios for the palm species *Hyphaene compressa*. The first panel of maps shows the SPATIAL and CLIMATE models. The SPATIAL model is based on six climatic predictor supplemented with six spatial filters converted to binary output using the threshold equal training sensitivity and specificity which corresponds to the 0 km dispersal mask (blue area). The 100 km dispersal scenario is derived by adding a 100 km buffer to the binary output of the SPATIAL model. The CLIMATE model is based on the same six climatic predictors and is depicted with climate suitability ranging from 0-1 with darker blue indicating higher suitability (see Methods; Supplementary Methods). The central panel shows the predicted suitability for the baseline time period (Current suitability) and for the end of the century (Future suitability) within the 0 km (white hatched area) and 100 km dispersal masks. The right panel shows the change in climatic suitability between the Current and Future scenarios and is computed as Current suitability minus Future suitability in a cell-by-cell manner. Negative values indicate a loss in climatic suitability. The individual maps were created in ArcGIS 10.2.2 (ESRI, Redlands, CA, USA).

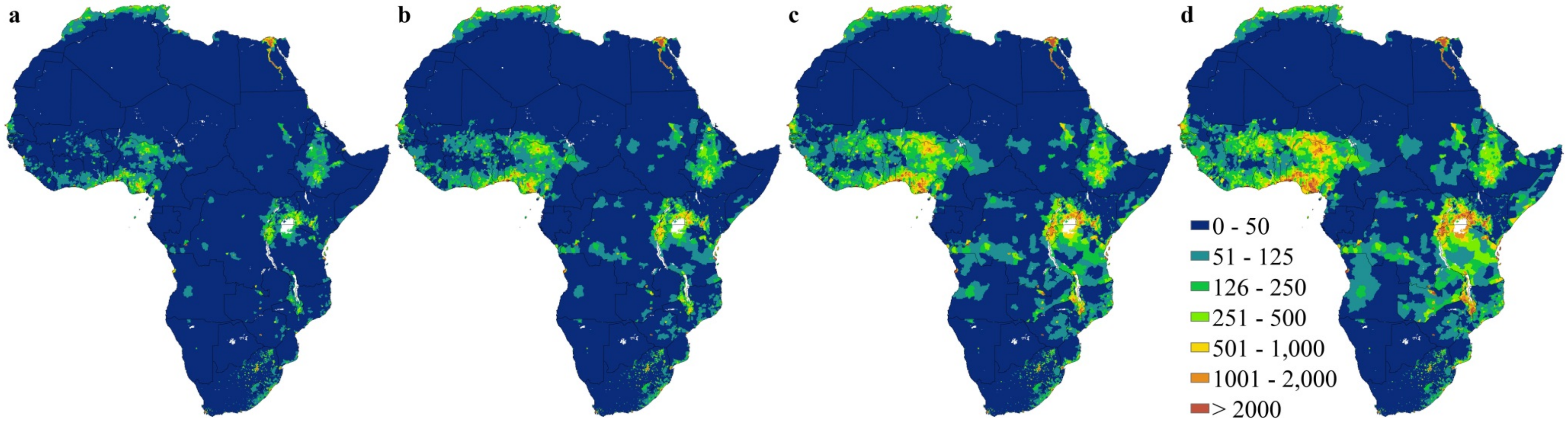

Figure S11 | Spatial patterns of human population density (people/km^2) across Africa. a, Gridded population density for year 2000. **b**, Gridded population density projected for year 2020. **c**, Gridded population density projected for year 2050 **d**, Gridded population density projected for year 2080**.** The map was created in ArcGIS 9.3 (ESRI, Redlands, CA, USA).

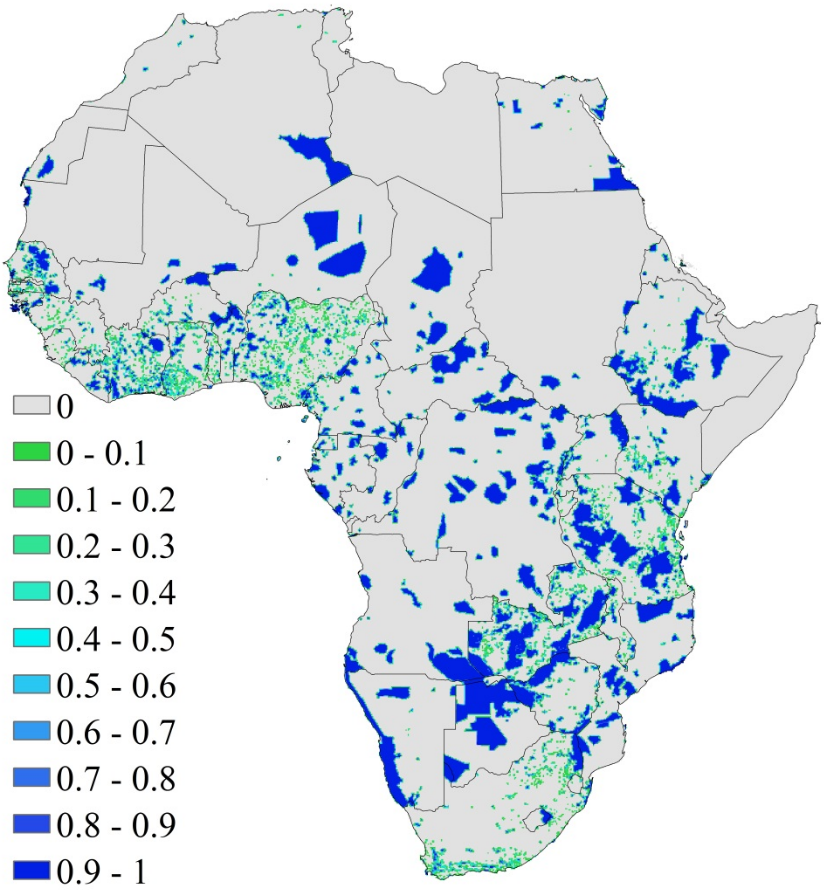

Figure S12 | Proportion of 10 × 10 km grids covered by protected areas across Africa. The map was created in ArcGIS 9.3 (ESRI, Redlands, CA, USA).

Supplementary Tables

Table S1 | Details on African palm species. Overview of 40 species (82.5% of total species recognized for Africa) extracted from the African palm database giving information of unique occurrence records available for each species at 10 × 10 km resolution. The habitat affiliation is given for each species. “Rainforest” includes species with strictly tropical rain forest affinity whereas “open-habitat” includes species which only occur in open habitats covering savanna woodlands to dry deserts. Two species can occur in both habitats and are marked “Mixed”.

Species	Habitat	No. of unique occurrences
Borassus aethiopum	Open-habitat	116
Borassus akeassii	Open-habitat	13
Calamus deerratus	Rainforest	140
Chamaerops humilis	Open-habitat	19
Elaeis guineensis	Mixed	113
Eremospatha cabrae	Rainforest	33
Eremospatha cuspidata	Rainforest	19
Eremospatha haullevilleana	Rainforest	100
Eremospatha hookeri	Rainforest	39
Eremospatha laurentii	Rainforest	49
Eremospatha macrocarpa	Rainforest	105
Eremospatha wendlandiana	Rainforest	47
Hyphaene compressa	Open-habitat	41
Hyphaene coriacea	Open-habitat	47
Hyphaene guineensis	Open-habitat	18
Hyphaene petersiana	Open-habitat	66
Hyphaene thebaica	Open-habitat	48
Laccosperma acutiflorum	Rainforest	26
Laccosperma laeve	Rainforest	39
Laccosperma opacum	Rainforest	90
Laccosperma robustum	Rainforest	55
Laccosperma secundiflorum	Rainforest	100
Medemia argun	Open-habitat	14
Oncocalamus macrospathus	Rainforest	14
Oncocalamus mannii	Rainforest	31
Oncocalamus tuleyi	Rainforest	16
Phoenix reclinata	Mixed	294
Podococcus acaulis	Rainforest	25
Podococcus barteri	Rainforest	65
Raphia farinifera	Open-habitat	39
Raphia hookeri	Rainforest	58
Raphia mambillensis	Rainforest	13
Raphia palma-pinus	Rainforest	32
Raphia regalis	Rainforest	23
Raphia sese	Rainforest	10
Raphia sudanica	Open-habitat	31
Raphia vinifera	Rainforest	13
Sclerosperma mannii	Rainforest	43
Sclerosperma profizianum	Rainforest	11
Sclerosperma walkeri	Rainforest	11

Table S2 | Summary statistics of human population density (HPD) within land cover classes across Africa and for continental Africa. The land cover classes are given by the Land-cover map of Africa produced as a part of the Global Land Cover project in 2000[40]. Mean, median and standard deviation of HPD has been extracted using zonal statistics in GIS at 1 × 1-km resolution.

Land cover class	Mean HPD	Median HPD	Standard deviation HPD
Cities	1606.12	667	2616.38
Irrigated croplands	676.81	130	863.17
Tree crops	399.13	326	403.60
Mangrove	*152.09*	*51*	*365.07*
Montane forest (>1500 m)	*100.67*	*65*	*134.99*
Waterbodies	89.42	29	319.31
Mosaic Forest / Croplands	75.29	37	154.02
Croplands (>50%)	68.49	32	165.59
Degraded evergreen lowland forest	63.61	35	106.30
**Continental Africa (CON-HPD)**	**50.14**	**34**	**57.88**
Open deciduous shrubland	37.37	10	130.87
Croplands with open woody vegetation	*35.98*	*19*	*62.85*
Submontane forest (900 -1500 m)	34.97	13	51.14
Deciduous shrubland with sparse trees	34.63	16	78.97
Closed deciduous forest	31.00	11	120.24
Deciduous woodland	29.19	13	95.99
Closed grassland	25.62	6	86.58
Swamp bushland and grassland	23.77	15	38.82
Mosaic Forest / Savanna	20.77	8	54.69
Open grassland with sparse shrubs	20.76	5	67.88
Salt hardpans	19.24	3	68.26
Sparse grassland	17.94	6	42.03
Closed evergreen lowland forest	16.36	6	57.76
Open grassland	10.12	3	28.34
Bare rock	8.72	0	82.07
Swamp forest	7.76	3	11.97
Stony desert	4.42	0	155.29
Sandy desert and dunes	1.40	0	14.57

HPD is given in number of people per km^2. The columns have been sorted in descending order based on Mean HPD. The year 2000 continental-wide measures of average, median and standard deviation of HPD across Africa are inserted and marked in **bold**. The mean is used as a threshold for high HPD in the analyses. Deviations from the expectation of HPD below CON-HPD in natural land cover classes and HPD above CON-HPD in human-influenced land cover classes are marked in italic and underlined.

Table S3 | Number of palm species projected to gain (winners; W) or lose (losers; L) climatic suitability within the protected area network. Loser and winner species for all, rainforest and open-habitat palms derived for three future time periods (2020, 2050, 2080) for different combinations of species distribution models (Maxent and Generalized Boosting Models), global circulation models (CCCMA; CSIRO; HADCM) and gas emission scenarios (A1B; A2A; B2A) for two CO_2-scenarios (+CO_2; −CO_2; see Supplementary Methods) within the margins of two dispersal scenarios (no-dispersal; 100-km-dispersal). Winner and loser species within the 100-km-dispersal mask are marked in bold.

		MAXENT																	
	CO_2-	CCCMA						CSIRO						HADCM					
Time	scenario	A1b		A2a		B2a		A1b		A2a		B2a		A1b		A2a		B2a	
ALL (40)		W	L	W	L	W	L	W	L	W	L	W	L	W	L	W	L	W	L
2020	+CO_2	17	23	23	17	16	24	17	23	13	27	13	27	16	24	18	22	18	22
		**20**	**20**	**28**	**12**	**21**	**19**	**19**	**21**	**14**	**26**	**16**	**24**	**19**	**21**	**21**	**19**	**12**	**18**
	−CO_2	13	27	22	18	17	23	14	26	12	28	12	28	14	26	17	23	16	24
		**18**	**22**	**23**	**17**	**18**	**22**	**18**	**22**	**13**	**27**	**15**	**25**	**18**	**22**	**19**	**21**	**19**	**21**
2050	+CO_2	10	30	17	23	14	26	13	27	11	29	13	27	11	29	10	30	16	24
		**12**	**28**	**20**	**20**	**19**	**21**	**16**	**24**	**13**	**27**	**13**	**27**	**13**	**27**	**12**	**28**	**17**	**23**
	−CO_2	7	33	14	26	12	28	9	31	10	30	11	29	9	31	8	32	13	27
		**10**	**30**	**16**	**24**	**16**	**24**	**15**	**25**	**13**	**27**	**13**	**27**	**11**	**29**	**9**	**31**	**15**	**25**
2080	+CO_2	6	34	8	32	15	25	5	35	7	33	10	30	6	34	7	33	10	30
		**8**	**32**	**11**	**29**	**17**	**23**	**5**	**35**	**10**	**30**	**13**	**27**	**7**	**33**	**7**	**33**	**13**	**27**
	−CO_2	6	34	6	34	12	28	4	36	5	35	10	30	6	34	6	34	8	32
		**6**	**34**	**7**	**33**	**13**	**27**	**6**	**34**	**4**	**36**	**8**	**32**	**6**	**34**	**7**	**33**	**10**	**30**
RAIN (27)																			
2020	+CO_2	10	17	15	12	10	17	8	19	6	21	7	20	7	20	11	16	10	17
		**10**	**17**	**19**	**8**	**13**	**14**	**9**	**18**	**6**	**21**	**7**	**20**	**9**	**18**	**12**	**15**	**13**	**14**
	−CO_2	7	20	15	12	9	18	9	18	6	21	7	20	9	18	8	19	9	18
		**9**	**18**	**16**	**11**	**11**	**16**	**8**	**19**	**6**	**21**	**7**	**20**	**8**	**19**	**10**	**17**	**11**	**16**
2050	+CO_2	5	22	11	16	8	19	6	21	6	21	6	21	5	22	5	22	9	18
		**7**	**20**	**12**	**15**	**11**	**16**	**8**	**19**	**6**	**21**	**6**	**21**	**5**	**22**	**6**	**21**	**10**	**17**
	−CO_2	5	22	8	19	6	21	5	22	5	22	6	21	5	22	4	23	8	19
		**6**	**21**	**10**	**17**	**10**	**17**	**7**	**20**	**6**	**21**	**6**	**21**	**7**	**20**	**5**	**22**	**8**	**19**
2080	+CO_2	5	22	5	22	9	18	3	24	5	22	5	22	4	23	4	23	5	22
		**6**	**21**	**7**	**20**	**10**	**17**	**3**	**24**	**6**	**21**	**6**	**21**	**4**	**23**	**4**	**23**	**7**	**20**
	−CO_2	5	22	5	22	6	21	3	24	3	24	5	22	3	24	4	23	5	22
		**5**	**22**	**5**	**22**	**7**	**20**	**4**	**23**	**3**	**24**	**5**	**22**	**4**	**23**	**4**	**23**	**5**	**22**
OPEN (11)																			
2020	+CO_2	6	5	6	5	6	5	6	5	7	4	5	6	7	4	8	3	7	4
		**9**	**2**	**7**	**4**	**7**	**4**	**9**	**2**	**7**	**4**	**8**	**3**	**9**	**2**	**8**	**3**	**8**	**3**
	−CO_2	5	6	5	6	6	5	7	4	6	5	5	6	6	5	6	5	6	5
		**8**	**3**	**6**	**5**	**6**	**5**	**9**	**2**	**6**	**5**	**7**	**4**	**9**	**2**	**8**	**3**	**7**	**4**
2050	+CO_2	4	7	5	6	5	6	5	6	5	6	6	5	5	6	5	6	6	5
		**4**	**7**	**7**	**4**	**7**	**4**	**6**	**5**	**6**	**5**	**6**	**5**	**5**	**6**	**6**	**5**	**6**	**5**
	−CO_2	2	9	5	6	5	6	3	8	5	6	5	6	4	7	4	7	5	6
		**3**	**8**	**5**	**6**	**5**	**6**	**6**	**5**	**6**	**5**	**6**	**5**	**5**	**6**	**4**	**7**	**6**	**5**
2080	+CO_2	1	10	3	8	5	6	1	10	2	9	5	6	2	9	3	8	5	6
		**1**	**10**	**3**	**8**	**6**	**5**	**2**	**9**	**4**	**7**	**6**	**5**	**3**	**8**	**3**	**8**	**5**	**6**
	−CO_2	1	10	1	10	5	6	2	9	2	9	5	6	3	8	2	9	3	8
		**1**	**10**	**2**	**9**	**5**	**6**	**2**	**9**	**1**	**10**	**3**	**8**	**2**	**9**	**3**	**8**	**5**	**6**

		GENERALISED BOOSTING METHOD																	
	CO_2-	CCCMA						CSIRO						HADCM					
Time	scenario	A1b		A2a		B2a		A1b		A2a		B2a		A1b		A2a		B2a	
ALL (40)		W	L	W	L	W	L	W	L	W	L	W	L	W	L	W	L	W	L
2020	+CO_2	6	34	12	28	9	31	6	34	6	34	7	33	7	33	8	32	6	34
		**10**	**30**	**13**	**27**	**11**	**29**	**10**	**30**	**8**	**32**	**9**	**31**	**8**	**32**	**9**	**31**	**10**	**30**
	−CO_2	6	34	12	28	9	31	6	34	6	34	7	33	6	34	8	32	6	34
		**9**	**31**	**13**	**27**	**11**	**29**	**8**	**32**	**7**	**33**	**9**	**31**	**8**	**32**	**9**	**31**	**9**	**31**
2050	+CO_2	2	38	6	34	3	37	1	39	3	37	3	37	2	38	3	37	3	37
		**3**	**37**	**10**	**30**	**8**	**32**	**2**	**38**	**5**	**35**	**6**	**34**	**3**	**37**	**3**	**37**	**5**	**35**
	−CO_2	2	38	3	37	3	37	0	40	3	37	3	37	2	38	3	37	4	36
		**3**	**37**	**9**	**31**	**6**	**34**	**2**	**38**	**5**	**35**	**5**	**35**	**3**	**37**	**3**	**37**	**5**	**35**
2080	+CO_2	0	40	2	38	4	36	0	40	2	38	2	38	1	39	2	38	3	37
		**2**	**38**	**2**	**38**	**7**	**33**	**1**	**39**	**4**	**36**	**5**	**35**	**2**	**38**	**2**	**38**	**4**	**36**
	−CO_2	0	40	2	38	4	36	0	40	2	38	2	38	1	39	2	38	3	37
		**2**	**38**	**2**	**38**	**6**	**34**	**1**	**39**	**3**	**37**	**4**	**36**	**1**	**39**	**2**	**38**	**4**	**36**
RAIN (27)																			
2020	+CO_2	4	23	10	17	7	20	4	23	4	23	5	22	4	23	6	21	5	22
		**7**	**20**	**11**	**16**	**9**	**18**	**7**	**20**	**6**	**21**	**7**	**20**	**5**	**22**	**7**	**20**	**8**	**19**
	−CO_2	4	23	10	17	7	20	4	23	4	23	5	22	4	23	6	21	5	22
		**6**	**21**	**11**	**16**	**9**	**18**	**5**	**22**	**5**	**22**	**7**	**20**	**5**	**22**	**7**	**20**	**7**	**20**
2050	+CO_2	1	26	2	25	2	25	0	27	2	25	2	25	1	26	2	25	2	25
		**2**	**25**	**8**	**19**	**6**	**21**	**1**	**26**	**4**	**23**	**4**	**23**	**2**	**25**	**2**	**25**	**3**	**24**
	−CO_2	1	26	5	22	2	25	1	26	2	25	2	25	1	26	2	25	3	24
		**2**	**25**	**7**	**20**	**4**	**23**	**1**	**26**	**4**	**23**	**3**	**24**	**2**	**25**	**2**	**25**	**3**	**24**
2080	+CO_2	0	27	1	26	3	24	0	27	1	26	1	26	0	27	1	26	2	25
		**1**	**26**	**1**	**26**	**6**	**21**	**0**	**27**	**3**	**24**	**3**	**24**	**1**	**26**	**1**	**26**	**3**	**24**
	−CO_2	0	27	1	26	3	24	0	27	1	26	1	26	0	27	1	26	2	25
		**1**	**26**	**1**	**26**	**5**	**22**	**0**	**27**	**2**	**25**	**3**	**24**	**0**	**27**	**1**	**26**	**3**	**24**
OPEN (11)																			
2020	+CO_2	2	9	2	9	2	9	2	9	2	9	2	9	3	8	2	9	1	10
		**3**	**8**	**2**	**9**	**2**	**9**	**3**	**8**	**2**	**9**	**2**	**9**	**3**	**8**	**2**	**9**	**2**	**9**
	−CO_2	2	9	2	9	2	9	2	9	2	9	2	9	2	9	2	9	1	10
		**3**	**8**	**2**	**9**	**2**	**9**	**3**	**8**	**2**	**9**	**2**	**9**	**3**	**8**	**2**	**9**	**2**	**9**
2050	+CO_2	1	10	1	10	1	10	0	11	1	10	1	10	1	10	1	10	1	10
		**1**	**10**	**2**	**9**	**2**	**9**	**1**	**10**	**1**	**10**	**2**	**9**	**1**	**10**	**1**	**10**	**2**	**9**
	−CO_2	1	10	1	10	1	10	0	11	1	10	1	10	1	10	1	10	1	10
		**1**	**10**	**2**	**9**	**2**	**9**	**1**	**10**	**1**	**10**	**2**	**9**	**1**	**10**	**1**	**10**	**2**	**9**
2080	+CO_2	0	11	1	10	1	10	0	11	1	10	1	10	1	10	1	10	1	10
		**1**	**10**	**1**	**10**	**1**	**10**	**1**	**10**	**1**	**10**	**2**	**9**	**1**	**10**	**1**	**10**	**1**	**10**
	−CO_2	0	11	1	10	1	10	0	11	1	10	1	10	1	10	1	10	1	10
		**1**	**10**	**1**	**10**	**1**	**10**	**1**	**10**	**1**	**10**	**1**	**10**	**1**	**10**	**1**	**10**	**1**	**10**

Table S4 | Dependent and independent variables used to build linear equations. Correction factors (C1−C4) for potential evapotranspiration used as dependent variables and CO_2 concentration rises as independent variables to build linear regression equations to be used to compute correction factors across a set of future CO_2 concentration rises (see supplementary Table S5) for the rescaling of future potential evapotranspiration. Correction factors marked in **bold** are derived from Kruijt *et al.*[9] for two different CO_2 concentration rises between the baseline and future conditions. The remaining correction factors (C1−C4 =1) are given presuming potential evapotranspiration is unchanged under stable CO_2 levels.

CO_2 rise (ppm)	C1	C2	C3	C4
0	1	1	1	1
150	**0.98**	**0.96**	**0.97**	**0.96**
385	**0.95**	**0.91**	**0.92**	**0.89**

Table S5 | Correction factors to rescale potential evapotranspiration (PET). Correction factors (c-factors) for all possible CO_2 concentration rises of Bern and ISAM models[10] across three gas emission scenarios (SRES; A1B, A2A, B2A) computed using linear regression equations (see below table) based on data derived from Kruijt *et al.*[9] (Supplementary Table S4, Fig. S7) and CO_2 concentration rises calculated as the difference in CO_2 between baseline (1970-1990 average) and three future time periods 2020 (average of 2010−2040), 2050 (2040−2070) and 2080 (2070−2100). Correction factors used to rescale PET are marked in bold.

Year	SRES model	[CO_2] rise (ppm)	C1	C2	C3	C4	Average (C1−C4)	C-factors (Average of Bern and ISAM
2020	B2 Bern	78	0.992	0.9824	0.9849	0.9777	0.98425	
	B2 ISAM	81.17	0.991683	0.981766	0.984266	0.976749	0.983616	**0.983933**
	A1 Bern	96	0.9902	0.9788	0.9813	0.9723	0.98065	
	A1 ISAM	100.67	0.989733	0.977866	0.980366	0.970899	0.979716	**0.980183**
	A2 Bern	93.25	0.990475	0.97935	0.98185	0.973125	0.9812	
	A2 ISAM	98.67	0.989933	0.978266	0.980766	0.971499	0.980116	**0.980658**
2050	B2 Bern	148	0.985	0.9684	0.9709	0.9567	0.97025	
	B2 ISAM	153.17	0.984483	0.967366	0.969866	0.955149	0.969216	**0.969733**
	A1 Bern	204.25	0.979375	0.95715	0.95965	0.939825	0.959	
	A1 ISAM	213.17	0.978483	0.955366	0.957866	0.937149	0.957216	**0.958108**
	A2 Bern	209.75	0.978825	0.95605	0.95855	0.938175	0.9579	
	A2 ISAM	220.92	0.977708	0.953816	0.956316	0.934824	0.955666	**0.956783**
2080	B2 Bern	229	0.9769	0.9522	0.9547	0.9324	0.95405	
	B2 ISAM	236.67	0.976133	0.950666	0.953166	0.930099	0.952516	**0.953283**
	A1 Bern	316.25	0.968175	0.93475	0.93725	0.906225	0.9366	
	A1 ISAM	327.17	0.967083	0.932566	0.935066	0.902949	0.934416	**0.935508**
	A2 Bern	385	0.9613	0.921	0.9235	0.8856	0.92285	
	A2 ISAM	401.67	0.959633	0.917666	0.920166	0.880599	0.919516	**0.921183**

Linear regressions are given by: C1 = y = -0.0001x + 0.9998; C2 = y = -0.0002x + 0.998; C3 = y = -0.0002x + 1.0005; C4 = y = -0.0003x + 1.0011 (see Fig S7).

Table S6 | Summary statistics of predictive ability among species distribution models (Maxent and Generalised Boosting Model) and threshold statistics for 40 palm species. Predictive ability is given by two measures namely the Area Under the receiver operating Curve (AUC) and the True Skill Statistics (TSS). Mean, median, min and max values are given for Maxent and Generalised Boosting Model (GBM) for both the SPATIAL (upper value) and CLIMATE (lower value) models (see Supplementary Methods) as well as for the threshold equal training sensitivity and specificity used for the SPATIAL model.

Species	AUC (SPATIAL/CLIMATE)						TSS (SPATIAL/CLIMATE)						Thresholds (SPATIAL)			
	Mean		Median		[min-max]		Mean		Median		[min-max]		Mean/Median		[min-max]	
	Maxent	GBM	Maxent	GBM	Maxent	GBM	Maxent	GBM	Maxent	GBM	Maxent	GBM	Maxent	GBM	Maxent	GBM
Borassus aethiopum	0.919/ 0.881	0.904/ 0.883	0.922/ 0.871	0.901/ 0.883	[0.892-0.946]/ [0.845-0.930]	[0.836-0.948]/ [0.847-0.910]	0.733/ 0.669	0.688/ 0.689	0.733/ 0.661	0.665/ 0.689	[0.657-0.804]/ [0.596-0.768]	[0.591-0.804]/ [0.602-0.773]	0.288/ 0.290	0.500/ 0.503	[0.263-0.307]	[0.453-0.524]
Borassus akeassii	0.981/ 0.957	0.945/ 0.771	0.989/ 0.965	0.959/ 0.817	[0.955-0.998]/ [0.890-0.992]	[0.733-1.000]/ [0.421-0.964]	0.966/ 0.937	0.635/ 0.557	0.968/ 0.953	0.499/ 0.490	[0.911-0.997]/ [0.836-0.987]	[0.000-1.000]/ [0.006-0.930]	0.288/ 0.303	0.189/ 0.145	[0.203-0.327]	[0.087-0.529]
Calamus deerratus	0.936/ 0.910	0.951/ 0.918	0.934/ 0.908	0.949/ 0.923	[0.923-0.953]/ [0.889-0.938]	[0.943-0.966]/ [0.895-0.935]	0.777/ 0.712	0.829/ 0.739	0.775/ 0.697	0.828/ 0.742	[0.752-0.819]/ [0.682-0.772]	[0.789-0.885]/ [0.648-0.804]	0.307/ 0.311	0.527/ 0.527	[0.247-0.343]	[0.490-0.568]
Chamaerops humilis	0.995/ 0.995	0.963/ 0.959	0.994/ 0.995	0.997/ 0.993	[0.992-0.998]/ [0.989-0.999]	[0.828-0.999]/ [0.825-0.997]	0.989/ 0.990	0.896/ 0.891	0.986/ 0.991	0.994/ 0.990	[0.983-0.996]/ [0.973-0.997]	[0.658-0.999]/ [0.657-0.994]	0.337/ 0.339	0.373/ 0.456	[0.288-0.372]	[0.054-0.587]
Elaeis guineensis	0.944/ 0.911	0.959/ 0.920	0.944/ 0.910	0.965/ 0.927	[0.911-0.964]/ [0.879-0.942]	[0.892-0.979]/ [0.801-0.977]	0.880/ 0.898	0.882/ 0.810	0.923/ 0.902	0.914/ 0.860	[0.748-0.955]/ [0.789-0.959]	[0.603-0.951]/ [0.526-0.963]	0.264/ 0.259	0.250/ 0.215	[0.224-0.328]	[0.120-0.484]
Eremospatha cabrae	0.957/ 0.956	0.906/ 0.875	0.963/ 0.955	0.967/ 0.883	[0.916-0.978]/ [0.942-0.976]	[0.547-0.991]/ [0.636-0.987]	0.930/ 0.786	0.831/ 0.762	0.974/ 0.753	0.928/ 0.765	[0.746-0.992]/ [0.615-0.992]	[0.327-0.984]/ [0.548-0.974]	0.323/ 0.323	0.255/ 0.257	[0.262-0.407]	[0.151-0.393]
Eremospatha cuspidata	0.972/ 0.904	0.934/ 0.880	0.985/ 0.902	0.934/ 0.877	[0.907-0.997]/ [0.803-0.995]	[0.889-0.964]/ [0.832-0.925]	0.813/ 0.719	0.773/ 0.645	0.808/ 0.710	0.777/ 0.641	[0.749-0.875]/ [0.660-0.791]	[0.710-0.886]/ [0.604-0.713]	0.143/ 0.146	0.513/ 0.512	[]0.108-0.174	[0.482-0.544]
Eremospatha haullevilleana	0.934/ 0.931	0.939/ 0.936	0.932/ 0.937	0.939/ 0.937	[0.919-0.956]/ [0.895-0.949]	[0.899-0.962]/ [0.925-0.952]	0.797/ 0.784	0.838/ 0.828	0.799/ 0.787	0.848/ 0.828	[0.711-0.861]/ [0.694-0.854]	[0.776-0.894]/ [0.797-0.861]	0.375/ 0.372	0.590/ 0.592	[0.317-0.420]	[0.537-0.651]
Eremospatha hookeri	0.990/ 0.982	0.986/ 0.950	0.990/ 0.985	0.988/ 0.975	[0.981-0.995]/ [0.959-0.996]	[0.962-0.992]/ [0.830-0.992]	0.966/ 0.955	0.959/ 0.877	0.968/ 0.966	0.966/ 0.914	[0.929-0.986]/ [0.882-0.983]	[0.906-0.977]/ [0.670-0.983]	0.149/ 0.152	0.265/ 0.273	[0.109-0.191]	[0.086-0.367]
Eremospatha laurentii	0.973/ 0.968	0.973/ 0.941	0.973/ 0.970	0.974/ 0.958	[0.963-0.983]/ [0.950-0.984]	[0.963-0.980]/ [0.847-0.978]	0.911/ 0.898	0.930/ 0.860	0.906/ 0.897	0.926/ 0.874	[0.892-0.943]/ [0.846-0.954]	[0.908-0.961]/ [0.712-0.934]	0.147/ 0.136	0.283/ 0.273	[0.120-0.199]	[0.243-0.343]
Eremospatha macrocarpa	0.972/ 0.962	0.970/ 0.959	0.974/ 0.965	0.968/ 0.960	[0.954-0.981]/ [0.941-0.977]	[0.956-0.988]/ [0.934-0.979]	0.860/ 0.831	0.845/ 0.806	0.861/ 0.838	0.826/ 0.807	[0.809-0.917]/ [0.739-0.899]	[0.763-0.962]/ [0.724-0.873]	0.101/ 0.097	0.337 /0.331	[0.090-0.129]	[0.193-0.607]
Eremospatha wendlandiana	0.991/ 0.990	0.990/ 0.974	0.992/ 0.991	0.992/ 0.987	[0.987-0.994]/ [0.977-0.997]	[0.976-0.999]/ [0.919-0.996]	0.963/ 0.960	0.953/ 0.917	0.974/ 0.969	0.953/ 0.969	[0.911-0.979]/ [0.890-0.992]	[0.866-0.994]/ [0.686-0.991]	0.137/ 0.133	0.247/ 0.207	[0.115-0.165]	[0.079-0.534]
Hyphaene compressa	0.968/ 0.912	0.977/ 0.915	0.968/ 0.913	0.986/ 0.917	[0.955-0.989]/ [0.811-0.976]	[0.912-0.997]/ [0.868-0.963]	0.882/ 0.773	0.909/ 0.749	0.881/ 0.782	0.928/ 0.718	[0.832-0.970]/ [0.520-0.956]	[0.817-0.992]/ [0.642-0.918]	0.143/ 0.144	0.253/ 0.235	[0.095-0.180]	[0.067-0.643]
Hyphaene coriacea	0.994/ 0.976	0.973/ 0.970	0.994/ 0.980	0.976/ 0.970	[0.988-0.996]/ [0.944-0.991]	[0.919-0.996]/ [0.947-0.995]	0.979/ 0.909	0.896/ 0.874	0.987/ 0.937	0.896/ 0.864	[0.944-0.990]/ [0.800-0.967]	[0.758-0.988]/ [0.797-0.977]	0.117/ 0.122	0.188/ 0.176	[0.065-0.133]	[0.056-0.426]
Hyphaene guineensis	0.993/ 0.993	0.978/ 0.940	0.997/ 0.994	0.997/ 0.983	[0.975-0.999]/ [0.986-0.998]	[0.828-1.000]/ [0.657-1.000]	0.983/ 0.988	0.928/ 0.827	0.996/ 0.990	0.997/ 0.827	[0.927-0.998]/ [0.970-0.998]	[0.650-0.999]/ [0.614-1.000]	0.189/ 0.125	0.407/ 0.439	[0.114-0.519]	[0.051-0.581]
Hyphaene petersiana	0.961/ 0.956	0.969/ 0.953	0.967/ 0.966	0.970/ 0.959	[0.944-0.973]/ [0.905-0.980]	[0.947-0.981]/ [0.918-0.982]	0.864/ 0.846	0.864/ 0.832	0.859/ 0.866	0.866/ 0.833	[0.802-0.939]/ [0.686-0.946]	[0.780-0.942]/ [0.739-0.909]	0.244/ 0.252	0.324/ 0.309	[0.187-0.301]	[0.119-0.461]
Hyphaene thebaica	0.842/ 0.763	0.849/ 0.778	0.842/ 0.765	0.855/ 0.778	[0.780-0.903]/ [0.647-0.869]	[0.775-0.915]/ [0.672-0.862]	0.618/ 0.536	0.637/ 0.530	0.595/ 0.532	0.630/ 0.528	[0.555-0.774]/ [0.415-0.713]	[0.470-0.812]/ [0.369-0.668]	0.416/ 0.417	0.396/ 0.395	[0.366-0.442]	[0.280-0.503]
Laccosperma acutiflorum	0.965/ 0.987	0.969/ 0.954	0.966/ 0.991	0.987/ 0.992	[0.884-0.996]/ [0.948-0.994]	[0.781-0.996]/ [0.645-0.996]	0.881/ 0.957	0.939/ 0.913	0.844/ 0.970	0.956/ 0.977	[0.770-0.988]/ [0.809-0.986]	[0.714-0.990]/ [0.590-0.993]	0.134/ 0.122	0.151/ 0.142	[0.090-0.210]	[0.075-0.246]
Laccosperma laeve	0.989/ 0.984	0.976/ 0.981	0.989/ 0.985	0.990/ 0.982	[0.982-0.995]/ [0.973-0.994]	[0.842-0.995]/ [0.966-0.994]	0.967/ 0.948	0.945/ 0.941	0.970/ 0.955	0.970/ 0.956	[0.939-0.985]/ [0.901-0.985]	[0.702-0.990]/ [0.886-0.975]	0.131/ 0.113	0.205/ 0.194	[0.105-0.194]	[0.059-0.418]
Laccosperma opacum	0.980/ 0.961	0.979/ 0.964	0.979/ 0.965	0.979/ 0.964	[0.974-0.989]/ [0.947-0.970]	[0.973-0.988]/ [0.949-0.978]	0.904/ 0.844	0.914/ 0.861	0.904/ 0.847	0.901/ 0.861	[0.867-0.954]/ [0.780-0.900]	[0.874-0.957]/ [0.816-0.929]	0.138/ 0.133	0.405/ 0.404	[0.118-0.162]	[0.291-0.487]
Laccosperma robustum	0.972/ 0.976	0.980/ 0.945	0.970/ 0.976	0.979/ 0.953	[0.947-0.995]/ [0.962-0.989]	[0.966-0.996]/ [0.893-0.973]	0.889/ 0.914	0.911/ 0.827	0.895/ 0.922	0.910/ 0.844	[0.780-0.984]/ [0.838-0.954]	[0.864-0.986]/ [0.718-0.930]	0.115/ 0.116	0.301/ 0.267	[0.079-0.148]	[0.162-0.594]

Species	AUC (SPATIAL/CLIMATE)						TSS (SPATIAL/CLIMATE)						Thresholds (SPATIAL)			
	Mean		Median		[min-max]		Mean		Median		[min-max]		Mean/Median		[min-max]	
	Maxent	GBM	Maxent	GBM	Maxent	GBM	Maxent	GBM	Maxent	GBM	Maxent	GBM	Maxent	GBM	Maxent	GBM
Laccosperma secundiflorum	0.966/ 0.951	0.965/ 0.950	0.968/ 0.954	0.968/ 0.950	[0.952-0.975]/ [0.928-0.963]	[0.935-0.977]/ [0.927-0.971]	0.877/ 0.826	0.870/ 0.806	0.877/ 0.831	0.881/ 0.806	[0.839-0.914]/ [0.757-0.875]	[0.766-0.910]/ [0.700-0.894]	0.195/ 0.190	0.459/ 0.487	[0.177-0.226]	[0.277-0.537]
Medemia argun	0.998/ 0.983	0.741/ 0.914	0.998/ 0.987	0.756/ 0.981	[0.994-1.000]/ [0.936-0.998]	[0.005-0.998]/ [0.733-0.999]	0.877/ 0.969	0.499/ 0.834	0.877/ 0.979	0.499/ 0.966	[0.749-0.982]/ [0.872-0.997]	[0.000-0.998]/ [0.472-0.998]	0.517/ 0.524	0.211/ 0.168	[0.483-0.541]	[0.097-0.557]
Oncocalamus macrospathus	0.934/ 0.948	0.852/ 0.914	0.930/ 0.946	0.972/ 0.956	[0.879-0.987]/ [0.901-0.988]	[0.505-0.999]/ [0.527-0.997]	0.976/ 0.932	0.831/ 0.756	0.974/ 0.942	0.952/ 0.914	[0.941-0.996]/ [0.856-0.980]	[0.489-0.998]/ [0.444-0.994]	0.264/ 0.260	0.276/ 0.256	[0.198-0.349]	[0.156-0.539]
Oncocalamus mannii	0.993/ 0.991	0.959/ 0.984	0.993/ 0.993	0.971/ 0.984	[0.985-0.999]/ [0.979-0.999]	[0.894-0.997]/ [0.957-0.999]	0.997/ 0.969	0.833/ 0.920	0.997/ 0.969	0.832/ 0.902	[0.996-0.999]/ [0.932-0.996]	[0.661-0.988]/ [0.816-0.996]	0.066/ 0.063	0.102/ 0.068	[0.043-0.088]	[0.036-0.286]
Oncocalamus tuleyi	0.998/ 0.996	0.999/ 0.963	0.998/ 0.996	0.999/ 0.996	[0.997-0.999]/ [0.994-0.997]	[0.997-1.000]/ [0.829-0.999]	0.992/ 0.994	0.998/ 0.927	0.992/ 0.994	0.998/ 0.994	[0.989-0.996]/ [0.992-0.996]	[0.994-1.000]/ [0.657-0.998]	0.354/ 0.355	0.350/ 0.345	[0.252-0.412]	[0.118-0.614]
Phoenix reclinata	0.910/ 0.872	0.988/ 0.978	0.911/ 0.877	0.997/ 0.999	[0.878-0.930]/ [0.847-0.885]	[0.896-1.000]/ [0.896-0.999]	0.981/ 0.985	0.976/ 0.957	0.985/ 0.984	0.994/ 0.997	[0.962-0.990]/ [0.965-0.997]	[0.796-1.000]/ [0.798-0.999]	0.330/ 0.332	0.374/ 0.378	[0.318-0.338]	[0.038-0.595]
Podococcus acaulis	0.996/ 0.995	0.989/ 0.985	0.995/ 0.995	0.995/ 0.985	[0.995-0.998]/ [0.991-0.998]	[0.951-0.997]/ [0.971-0.993]	0.688/ 0.977	0.942/ 0.926	0.688/ 0.976	0.982/ 0.926	[0.603-0.745]/ [0.970-0.983]	[0.800-0.994]/ [0.861-0.972]	0.257/ 0.254	0.178/ 0.141	[0.196-0.317]	[0.034-0.617]
Podococcus barteri	0.994/ 0.992	0.911/ 0.883	0.994/ 0.992	0.911/ 0.887	[0.992-0.995]/ [0.988-0.994]	[0.893-0.934]/ [0.842-0.903]	0.880/ 0.606	0.705/ 0.630	0.865/ 0.602	0.695/ 0.642	[0.770-0.985]/ [0.565-0.647]	[0.668-0.770]/ [0.560-0.669]	0.154/ 0.156	0.574/ 0.574	[0.114-0.174]	[0.561-0.590]
Raphia farinifera	0.961/ 0.874	0.949/ 0.898	0.963/ 0.863	0.960/ 0.922	[0.931-0.996]/ [0.818-0.963]	[0.828-0.982]/ [0.715-0.986]	0.944/ 0.685	0.837/ 0.763	0.942/ 0.680	0.852/ 0.828	[0.916-0.976]/ [0.573-0.869]	[0.706-0.926]/ [0.372-0.952]	0.215/ 0.225	0.229/ 0.201	[0.146-0.281]	[0.119-0.350]
Raphia hookeri	0.985/ 0.972	0.979/ 0.967	0.985/ 0.972	0.988/ 0.972	[0.977-0.991]/ [0.951-0.988]	[0.902-0.990]/ [0.927-0.988]	0.945/ 0.895	0.956/ 0.887	0.965/ 0.893	0.960/ 0.900	[0.663-0.999]/ [0.801-0.978]	[0.887-0.974]/ [0.801-0.953]	0.106/ 0.108	0.371/ 0.368	[0.079-0.127]	[0.126-0.548]
Raphia mambillensis	0.964/ 0.958	0.853/ 0.890	0.989/ 0.974	0.994/ 0.998	[0.748-0.999]/ [0.914-0.992]	[0.496-1.000]/ [0.375-1.000]	0.975/ 0.923	0.844/ 0.843	0.981/ 0.965	0.988/ 0.998	[0.941-0.988]/ [0.833-0.987]	[0.486-1.000]/ [0.013-1.000]	0.155/ 0.179	0.287/ 0.240	[0.058-0.326]	[0.129-0.547]
Raphia palma-pinus	0.990/ 0.961	0.976/ 0.935	0.990/ 0.972	0.984/ 0.940	[0.982-0.993]/ [0.891-0.990]	[0.908-0.996]/ [0.829-0.988]	0.969/ 0.889	0.879/ 0.792	0.963/ 0.947	0.879/ 0.792	[0.942-0.997]/ [0.644-0.975]	[0.652-0.986]/ [0.313-0.968]	0.141/ 0.111	0.156/ 0.116	[0.086-0.293]	[0.031-0.499]
Raphia regalis	0.987/ 0.986	0.956/ 0.962	0.987/ 0.986	0.991/ 0.983	[0.975-0.998]/ [0.971-0.998]	[0.753-0.997]/ [0.852-0.995]	0.950/ 0.965	0.931/ 0.900	0.970/ 0.968	0.973/ 0.943	[0.899-0.994]/ [0.919-0.996]	[0.725-0.995]/ [0.660-0.990]	0.102/ 0.093	0.250/ 0.184	[0.064-0.181]	[0.140-0.576]
Raphia sese	0.969/ 0.953	0.933/ 0.814	0.977/ 0.957	0.982/ 0.814	[0.939-0.997]/ [0.912-0.988]	[0.726-0.996]/ [0.549-0.988]	0.944/ 0.912	0.873/ 0.709	0.944/ 0.918	0.967/ 0.709	[0.891-0.964]/ [0.824-0.984]	[0.480-0.994]/ [0.439-0.982]	0.207/ 0.265	0.252/ 0.231	[0.124-0.265]	[0.121-0.463]
Raphia sudanica	0.974/ 0.932	0.901/ 0.862	0.975/ 0.940	0.905/ 0.832	[0.953-0.988]/ [0.888-0.957]	[0.764-0.988]/ [0.787-0.947]	0.996/ 0.862	0.831/ 0.756	0.997/ 0.868	0.819/ 0.719	[0.985-0.999]/ [0.794-0.907]	[0.644-0.967]/ [0.637-0.887]	0.398/ 0.389	0.204/ 0.203	[0.338-0.450]	[0.057-0.331]
Raphia vinifera	0.997/ 0.995	0.921/ 0.945	0.998/ 0.997	0.997/ 0.994	[0.992-0.999]/ [0.975-0.999]	[0.744-1.000]/ [0.522-0.999]	0.914/ 0.991	0.797/ 0.886	0.927/ 0.996	0.994/ 0.988	[0.812-0.966]/ [0.951-0.999]	[0.493-0.999]/ [0.455-0.998]	0.222/ 0.195	0.352/ 0.410	[0.154-0.432]	[0.076-0.573]
Sclerosperma mannii	0.977/ 0.963	0.950/ 0.962	0.977/ 0.970	0.974/ 0.962	[0.957-0.990]/ [0.922-0.983]	[0.863-0.992]/ [0.925-0.986]	0.905/ 0.883	0.891/ 0.847	0.905/ 0.907	0.922/ 0.860	[0.874-0.938]/ [0.752-0.944]	[0.676-0.965]/ [0.650-0.933]	0.196/ 0.190	0.256/ 0.257	[0.170-0.246]	[0.057-0.429]
Sclerosperma profizianum	0.919/ 0.892	0.920/ 0.917	0.921/ 0.899	0.965/ 0.956	[0.888-0.943]/ [0.805-0.939]	[0.488-0.987]/ [0.705-0.989]	0.928/ 0.871	0.852/ 0.810	0.920/ 0.876	0.933/ 0.928	[0.881-0.989]/ [0.768-0.917]	[0.000-0.981]/ [0.079-0.980]	0.470/ 0.471	0.348/ 0.371	[0.449-0.492]	[0.171-0.503]
Sclerosperma walkeri	0.948/ 0.923	0.761/ 0.812	0.941/ 0.928	0.741/ 0.964	[0.914-0.993]/ [0.878-0.960]	[0.259-0.998]/ [0.243-1.000]	0.997/ 0.892	0.585/ 0.686	0.998/ 0.895	0.500/ 0.930	[0.992-0.999]/ [0.861-0.943]	[0.000-0.996]/ [0.000-0.999]	0.343/ 0.341	0.245/ 0.221	[0.302-0.395]	[0.148-0.409]

References

1 Blach-Overgaard, A., Svenning, J. C. & Balslev, H. Climate change sensitivity of the African ivory nut palm,Hyphaene petersiana Klotzsch ex Mart. (Arecaceae) – a keystone species in SE Africa. *IOP C. Ser. Earth Env. Sci.* **8,** 012014 (2009).

2 Dransfield, J. *et al.* A new coryphoid palm genus from madagascar. *Bot. J. Linn. Soc.* **156,** 79-91 (2008).

3 Blach-Overgaard, A., Kissling, W. D., Dransfield, J., Balslev, H. & Svenning, J.-C. Multimillion-year climatic effects on palm species diversity in Africa. *Ecology* **94,** 2426-2435 (2013).

4 Tuley, P. *The Palms of Africa*. (The Trendrine Press, Zennor, St. Ives, Cornwall, Great Britain, 1995).

5 Hijmans, R. J., Cameron, S. E., Parra, J. L., Jones, P. G. & Jarvis, A. Very high resolution interpolated climate surfaces for global land areas. *Int. J. Climatol. 25, 1965-1978 (2005).*

6 Ramirez-Villegas, J. & Jarvis, A. *Downscaling Global Circulation Model Output: the Delta Method Decision and Policy Analysis Working Paper No. 1* (International Center for Tropical Agriculture, 2010). Available at: http://www.ccafs-climate.org/downloads/docs/Downscaling-WP-01.pdf. (Accessed: 1st June 2012).

7 Nakicenovic, N. *et al. Summary for policymakers. Special report on emissions scenarios. A special report of Working Group III of the Intergovernmental Panel on Climate Change*. (Cambridge University Press, Cambridge, United Kingdom, 2000).

8 Svenning, J.-C., Fløjgaard, C., Marske, K. A., Nógues-Bravo, D. & Normand, S. Applications of species distribution modeling to paleobiology. *Quat. Sci. Rev.* **30,** 2930-2947 (2011).

9 Kruijt, B., Witte, J.-P. M., Jacobs, C. M. J. & Kroon, T. Effects of rising atmospheric CO2 on evapotranspiration and soil moisture: A practical approach for the Netherlands. *J. Hydrol.* **349,** 257-267 (2008).

10 IPPC. *IPCC Climate Change 2001: The Scientific Basis. Contribution of Working Group I to the Third Assessment Report of the Intergovernmental Panel on Climate Change*. Edited by Houghton, J. T., et al. (Cambridge University Press, Cambridge, United Kingdom, 2001)

11 Thornthwaite, C. W. An approach toward a rational classification of climate. *Geogr. Rev.* **38,** 55-94 (1948).

12 Phillips, S. J., Anderson, R. P. & Schapire, R. E. Maximum entropy modeling of species geographic distributions. *Ecol. Model.* **190,** 231-259 (2006).

13 Ridgeway, G. gbm: generalized boosted regression models. R package version 1.6-3.1. (2010). URL: http://CRAN.R-project.org/package=gbm.

14 R Core Team. R: A language and environment for statistical computing (R Foundation for Statistical Computing, Wienna, Austria, 2012). URL: http://www.R-project.org/.

15 Thuiller, W., Lafourcade, B., Engler, R. & Araújo, M. B. BIOMOD - a platform for ensemble forecasting of species distributions. *Ecography* **32,** 369-373 (2009).

16 Elith, J., Kearney, M. & Phillips, S. The art of modelling range-shifting species. *Method. Ecol. Evol.* **1,** 330-342 (2010).

17 Elith, J. *et al.* A statistical explanation of MaxEnt for ecologists. *Divers. Distrib.* **17,** 43-57 (2011).

18 Elith, J., Graham, C. H., Wisz, M. S. & Zimmermann, N. E. Novel methods improve prediction of species' distributions from occurrence data. *Ecography* **29,** 129-151 (2006).

19 Elith, J. & Graham, C. H. Do they? How do they? WHY do they differ? On finding reasons for differing performances of species distribution models. *Ecography* **32,** 66-77 (2009).

20 De Marco, P., Diniz-Filho, J. A. & Bini, L. M. Spatial analysis improves species distribution modelling during range expansion. *Biol. Lett.* **4,** 577-580 (2008).

21 Blach-Overgaard, A., Svenning, J. C., Dransfield, J., Greve, M. & Balslev, H. Determinants of palm species distributions across Africa: the relative roles of climate, non-climatic environmental factors, and spatial constraints. *Ecography* **33,** 380-391 (2010).

22 Borcard, D. & Legendre, P. All-scale spatial analysis of ecological data by means of principal coordinates of neighbour matrices. *Ecol. Model.* **153,** 51-68 (2002).

23 Rangel, T. F., Diniz-Filho, J. A. F. & Bini, L. M. SAM: a comprehensive application for spatial analysis in macroecology. *Ecography* **33,** 46-50 (2010).

24 Phillips, S. J. & Dudik, M. Modeling of species distributions with Maxent: new extensions and a comprehensive evaluation. *Ecography* **31,** 161-175 (2008).

25 Wisz, M. S. & Guisan, A. Do pseudo-absence selection strategies influence species distribution models and their predictions? An information-theoretic approach based on simulated data. *BMC Ecol.* **9,** 8 (2009).

26 Lobo, J. M., Jiménez-Valverde, A. & Real, R. AUC: a misleading measure of the performance of predictive distribution models. *Global Ecol. Biogeogr.* **17,** 145-151 (2008).

27 Allouche, O., Tsoar, A. & Kadmon, R. Assessing the accuracy of species distribution models: prevalence, kappa and the true skill statistic (TSS). *J. Appl. Ecol.* **43,** 1223-1232 (2006).

28 Elith, J. in *Quantitative Methods in Conservation Biology* (eds Ferson, S. & Burgman, M. A.) Ch. 4, 39-58 (Springer-Verlag, New York, 2000).

29 Araujo, M. B., Thuiller, W. & Yoccoz, N. G. Reopening the climate envelope reveals macroscale associations with climate in European birds. *Proc. Natl. Acad. Sci. U.S.A.* **106,** E45-E46 (2009).

30 Watrin, J., Lézine, A.-M. & Hély, C. Plant migration and plant communities at the time of the "green Sahara". *Comptes Rendus Geosci.* **341,** 656-670 (2008).

31 Svenning, J. C. & Sandel, B. Disequilibrium vegetation dynamics under future climate change. *Am. J. Bot.* **100,** 1266-1286 (2013).

32 Thuiller, W., Lavorel, S., Araujo, M. B., Sykes, M. T. & Prentice, I. C. Climate change threats to plant diversity in Europe. *Proc. Natl. Acad. Sci. U.S.A.* **102,** 8245-8250 (2005).

33 Thomas, C. D. *et al.* Extinction risk from climate change. *Nature* **427,** 145-148 (2004).

34 Liu, C., Berry, P. M., Dawson, T. P. & Pearson, R. G. Selecting thresholds of occurrence in the prediction of species distributions. *Ecography* **28,** 385-393 (2005).

35 Nenzén, H. K. & Araújo, M. B. Choice of threshold alters projections of species range shifts under climate change. *Ecol. Model.* **222,** 3346-3354 (2011).

36 Hof, C., Araujo, M. B., Jetz, W. & Rahbek, C. Additive threats from pathogens, climate and land-use change for global amphibian diversity. *Nature* **480,** 516-519 (2011).

37 Beaumont, L. J., Hughes, L. & Pitman, A. J. Why is the choice of future climate scenarios for species distribution modelling important? *Ecol. Lett.* **11,** 1135-1146 (2008).

38 Center for International Earth Science Information Network - CIESIN - Columbia University & Centro Internacional de Agricultura Tropical - CIAT. (NASA Socioeconomic Data and Applications Center (SEDAC), Palisades, NY, 2005). Available at: http://dx.doi.org/10.7927/H4XK8CG2. (Accessed: 1st June 2012).

39 Rakotoarinivo, M., Dransfield, J., Bachman, S. P., Moat, J. & Baker, W. J. Comprehensive Red List assessment reveals exceptionally high extinction risk to Madagascar palms. *PloS One* **9,** e103684, (2014).

40 Mayaux, P., Bartholome, E., Fritz, S. & Belward, A. A new land-cover map of Africa for the year 2000. *J. Biogeogr.* **31,** 861-877 (2004).

41 IUCN and WDPA-WCMC. *The World Database on Protected Areas* (Cambridge, UK: UNEP-WCMC, 2009). Available at: http://www.wdpa.org. (Accessed: 1st June 2012).

42 Araujo, M. B., Alagador, D., Cabeza, M., Nogues-Bravo, D. & Thuiller, W. Climate change threatens European conservation areas. *Ecol. Lett.* **14,** 484-492 (2011).
